# Brainstem enkephalinergic neural circuit underlying cold-induced pain relief in mice

**DOI:** 10.1126/sciadv.aea3106

**Published:** 2026-11-06

**Authors:** Hayun Kim, Yoonkyung Lee, Seog Bae Oh

**Affiliations:** ^1^ Interdisciplinary Program in Neuroscience, Seoul National University, Seoul 08826, Republic of Korea.; ^2^ Department of Neurobiology and Physiology, School of Dentistry and Dental Research Institute, Seoul National University, Seoul 03080, Republic of Korea.; ^3^ ADA Forsyth Institute, Cambridge, MA, USA.

## Abstract

Application of cold or cold-mimicking chemicals to injury has long been recognized as an effective means of pain relief. However, underlying neural mechanisms remain elusive. Here, we identified a cold-responsive neuronal subset within the lateral parabrachial nucleus (lPBN), the thermosensory relay region in the hindbrain, that mediates cold-induced analgesia in mice. Activation of these neurons and their projection to ventrolateral periaqueductal gray (vlPAG) increased the nociceptive threshold, while ablation of these neurons attenuated analgesia induced by cold-mimicking chemicals. We further identified that these neurons express precursor gene of enkephalin (*Penk*), which is necessary for TRPM8 (transient receptor potential melastatin 8)–mediated pain relief. Mechanistically, cold-responsive lPBN neurons frequency-dependently released enkephalin peptide into vlPAG to activate the descending pain modulation circuitry via disinhibition. Activation of cold-responsive neurons reduced spinal cord responses to noxious stimuli, suggesting the involvement of a top-down pain modulation pathway. These findings propose a central mechanism underlying cold-induced pain relief, which could be a novel therapeutic target.

## INTRODUCTION

Cooling of the regions associated with pain has long been recognized as a method to induce pain relief (analgesia) (*1*–*6*). Historically, Hippocrates documented the use of cold water to alleviate joint pain, ulcers, and sprains ∼2400 years ago (*7*). Similarly, administration of a chemical agonist for transient receptor potential melastatin 8 (TRPM8), which is an essential ion channel involved in cooling perception (*8*, *9*), induces robust analgesia, mimicking cold-induced activation of the TRPM8 channel in peripheral sensory neurons. In contemporary clinical practice, over-the-counter creams and transdermal patches containing the TRPM8 agonists such as menthol or eucalyptol are widely used as the medication for pain management (*2*, *3*).

Such cold-induced, TRPM8-mediated analgesia has been attributed to peripheral mechanisms including decreased peripheral nerve conductance by physical cooling, desensitization of nociceptive nerve endings, and inactivation of voltage-gated calcium and/or sodium channels by menthol (*6*, *10*, *11*). Nevertheless, multiple lines of evidence suggest that cold-induced analgesia is primarily mediated by the central nervous system rather than by peripheral mechanisms. For instance, selective genetic ablation of cold-sensitive TRPM8^+^ fibers (sparing nociceptive neurons) abolished cold-induced analgesia (*12*). These results suggest that cold-induced analgesia does not originate from a direct inhibition of nociceptor activity upon cold application but rather involves an inhibition of nociceptive signaling by TRPM8^+^ fibers at the central level. In addition, pain relief induced by skin cooling and menthol is attenuated by the opioid receptor antagonist naloxone in both human subjects and preclinical mouse models, indicating the involvement of a central endogenous opioidergic pain modulation mechanism in cooling-induced analgesia (*1*, *4*, *13*). Moreover, cold-induced analgesia is not confined to the site of cold application; stimulation of distal or even contralateral regions similarly elicits marked analgesic effects (*14*, *15*).

Temperature information from the periphery is detected via thermosensory neurons and subsequently transmitted to the superficial dorsal horn of spinal cord and supraspinal regions for temperature information processing and representation (*16*). Among the supraspinal regions involved in temperature representation, the lateral parabrachial nucleus (lPBN) is well recognized as a first-order sensory gateway, integrating nociceptive and thermosensory signals including cold (*17*, *18*). Through various interoceptive and external signaling, lPBN also functions as a regulatory node for pain on the basis of homeostatic demands (*19*–*21*). Recent studies have highlighted the crucial role of the lPBN in pain regulation. For instance, activating a specific subset of lPBN neurons induces acute analgesia and reversed morphine-induced hyperalgesia (*22*, *23*). In particular, activation of the lPBN to the periaqueductal gray (PAG) neural circuit has been implicated in analgesic processes (*24*, *25*). Thus, it is plausible that the lPBN and its downstream projections might contribute to cold-induced analgesia as a top-down pain modulation mechanism. Collectively, these findings suggest that peripheral cold stimulation elicits analgesic effects, which are dominantly mediated by central nervous system pathways, extending beyond local or peripheral effects. However, despite the historical and clinical use of cold-induced analgesia, the central neural circuits and molecular mechanism underlying such an analgesic effect have been insufficiently understood.

To address this gap in knowledge, we used integrative approaches combining behavioral assays in a mouse model, chemogenetic activity modulation using permanent marking of active neural clusters through targeted recombination in active neural populations (FosTRAP), in vivo calcium imaging, ex vivo electrophysiology, and transcriptomic analysis to elucidate the circuit-to-molecular mechanisms of cold-induced analgesia. Notably, we identified a population of cold-responsive neurons within the lPBN (lPBN^cold^ neurons). lPBN^cold^ neurons were found to be necessary and sufficient for TRPM8-dependent analgesia, mediating the analgesic effect via projections to the ventrolateral PAG (vlPAG). Furthermore, activation of lPBN^cold^ neurons released enkephalin (a well-known antinociceptive neuropeptide) in a frequency-dependent manner to depolarize vlPAG projection neurons through disinhibition and diminished noxious stimulation–induced Fos expression in the spinal cord, suggesting the engagement of the descending inhibitory pathways. These findings provide insights into the neural circuits underlying cold-induced analgesia and highlight a critical role of the enkephalinergic lPBN-to-PAG neural circuit in TRPM8-dependent analgesia.

## RESULTS

### TRPM8-mediated antinociception and activation of supraspinal brain regions by cooling

To investigate whether cutaneous cooling elicits pain relief (analgesia) in mice model, we first assessed the effects of peripheral cold stimulation on mechanical pain sensitivity by exposing hindpaw to a 17°C metal plate for 5 min (fig. S1A). As expected, physically cooling the hindpaw induced substantial analgesia in the complete Freund’s adjuvant (CFA)–induced inflammatory pain model, in both mechanical pain and heat pain (fig. S1, B and C). Furthermore, to avoid the change of peripheral sensory nerve conductance under physical cooling and given the technical limitations of simultaneously applying physical cooling and conducting behavioral assays in the same hindpaw for a prolonged time period (*26*), we used topical application of potent TRPM8 agonist icilin (Fig. 1A), which mimics cooling of skin through activation of TRPM8-expressing fibers as a behavioral testing paradigm (*27*). Consistent with the previous report (*28*), topical icilin treatment to hindpaw increased the mechanical withdrawal threshold in both naïve and CFA-induced inflammatory pain models (Fig. 1, B and C). Furthermore, in concordance with the reports in human subjects (*3*, *14*, *15*), icilin treatment to distal regions (e.g., to the tail) resulted in an increased mechanical withdrawal threshold in the inflamed hindpaw (Fig. 1D). Given that TRPM8-induced analgesia has traditionally been attributed to the peripheral mechanism such as desensitization or inhibition of voltage-gated calcium channels (*6*, *10*), we first aimed to investigate the effect of TRPM8 activation within the peripheral nervous system. To assess this, we applied capsaicin to the primary culture of dorsal root ganglion (DRG) neurons to induce TRPV1 (transient receptor potential vanilloid 1)–dependent nociceptive neuron activation. We then tested whether concomitant icilin pretreatment could inhibit a capsaicin-induced calcium response through the direct peripheral mechanism. Our analysis revealed no significant difference in the calcium response to capsaicin treatment between vehicle-pretreated DRG neurons and icilin-pretreated DRG neurons (fig. S2). These findings suggest a lack of neuronal desensitization through TRPM8 activation, highlighting the involvement of the central nervous system but not the peripheral nervous system in cold-induced analgesia.

**Fig. 1.**
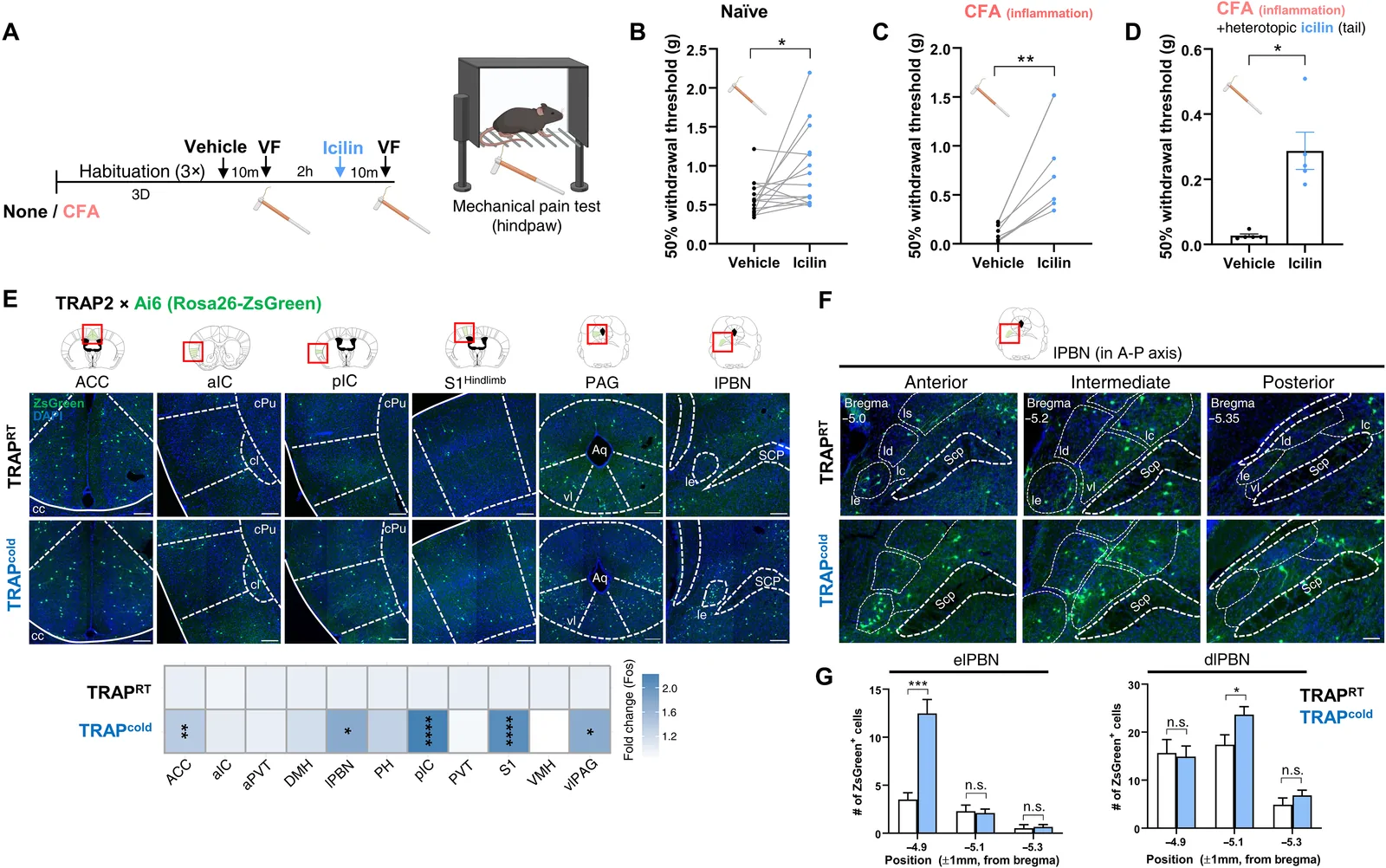
TRPM8-mediated antinociception and activation of supraspinal brain regions by cooling. (**A**) Experimental scheme and timeline for the icilin-induced pain relief experiment. m, minutes; h, hours. Created in BioRender. Oh (2026), https://biorender.com/wtbnd8f. (**B**) Mechanical sensitivity measure with 50% withdrawal thresholds in naïve mice in topical vehicle or icilin treatment. (**C**) Ipsilateral paw mechanical sensitivity measure with 50% withdrawal thresholds in CFA-induced inflammatory pain model mice in topical vehicle or icilin treatment. (**D**) Mechanical sensitivity measure with 50% withdrawal thresholds in naïve mice in topical vehicle or icilin treatment to the tail (distal) region. (**E**) Distribution of Zsg-positive neurons in a diverse brain region of an RT-TRAP mouse and cold-TRAP mouse (top) and quantification of Zsg-positive neurons per section (normalized to control; bottom). Green: ZsGreen; blue: DAPI. Scale bars, 100 μm. cPu, caudal putamen; cl, claustrum. (**F**) Distribution of Zsg-positive neurons of a control-TRAP mouse and cold-TRAP mouse in lPBN. Green: ZsGreen; blue: DAPI. Scale bar, 100 μm. Scp, superior cerebellar peduncle. (**G**) Quantification of Zsg-positive neurons in elPBN (left) and dlPBN (right). Data are presented as the means ± SEM. *P < 0.05, **P < 0.01, ***P < 0.001, and ****P < 0.0001. The number of mice and statistical tests are listed in table S1.

Considering prior evidence implicating supraspinal structures in cold-induced analgesia (*14*, *29*), we next sought to characterize the pattern of neuronal activation at the whole-brain level. To selectively label and further manipulate neurons responsive to peripheral cold stimulus, we used FosTRAP transgenic mice (Fos-iCre/ERT2) crossed with Ai6 reporter mice (TRAP2; Ai6). Consistent with previous reports (*30*, *31*), we observed robust peripheral cold stimulus–induced neuronal activation in the posterior insular cortex (pIC) and primary somatosensory cortex (S1), regions known to be activated by non-noxious cooling and TRPM8 agonist exposure. Notably, we also detected an increase in the number of labeled neurons within the lPBN (Fig. 1E), which has been suggested as a primary sensory hub for peripheral sensory stimuli (*32*). Thus, we focused on the functional involvement of lPBN in the cold-induced analgesia in the following experiments. Given the anatomical and functional heterogeneity of the lPBN, which consists of multiple subnuclei with distinctive molecular and functional profiles (*33*), we quantified cold-responsive neurons along the anterior-to-posterior axis and within the specific subdivisions of the lPBN. Notably, cold-responsive lPBN neurons were mainly located in the external lateral division of anterior lPBN (elPBN) and dorsal lateral division of intermediate lPBN (dlPBN) (Fig. 1, F and G), consistent with a prior report (*17*). To note, cold-responsive lPBN neurons showed little overlap with calcitonin gene-related peptide through the anterior-to-posterior axis (fig. S3), which constitutes a major population in elPBN (*33*).

### Response characteristics of lPBN neurons observed through in vivo calcium imaging

We performed in vivo microendoscopy to measure the neuronal calcium response in the lPBN to investigate the real-time response patterns of lPBN to the array of peripheral stimuli in vivo. The activity of lPBN neurons at a single-cell level was monitored during peripheral stimulation using heat (55°C hot water), noxious pinch, and cold (4°C cold water) applied to the tail region (Fig. 2, A to C), considering the lack of somatotopic arrangement in lPBN to peripheral stimulus (*34*, *35*). In vivo calcium imaging revealed distinct neuronal populations within the lPBN: One subset exhibited responsiveness to heat and noxious pinch but was not responsive to cold stimuli [hereafter referred to as lPBN^HP^ (heat-pinch) neurons], while another subset is being responsive to cold stimuli (lPBN^cold^ neurons) but not to heat and noxious pinch stimuli. A significant calcium response of lPBN^HP^ neurons, but not of lPBN^cold^ neurons, was observed 3.08 ± 0.19 s after the onset of prolonged heat exposure, corresponding to the average latency of the nocifensive tail-flick response observed in mice (Fig. 2, D and E, dashed line). Similarly, tail pinch with blunt forceps induced robust calcium responses in lPBN^HP^ neurons but not in lPBN^cold^ neurons (Fig. 2, D and F). Conversely, cold stimulation significantly increased the calcium response in lPBN^cold^ neurons while concomitantly decreasing the calcium response in lPBN^HP^ neurons (Fig. 2, D and G). To validate the functional segregation of lPBN neurons, we conducted double labeling with TRAP-Fos immunostaining (fig. S4A). Quantification of the TRAP method demonstrated a specificity of 0.50 and an efficiency of 0.35, further supporting the functional distinction between heat- and cold-responsive lPBN neuronal populations (fig. S4B; see table S1). As observed in the previous result, cold-TRAP neurons were primarily localized to the anterior elPBN and intermediate dlPBN, whereas hot-TRAP neurons were predominantly found in the posterior elPBN and intermediate dlPBN. However, cold- and hot-responsive dlPBN neurons shows non-overlapping distribution (fig. S4, C to E; see table S1).

**Fig. 2.**
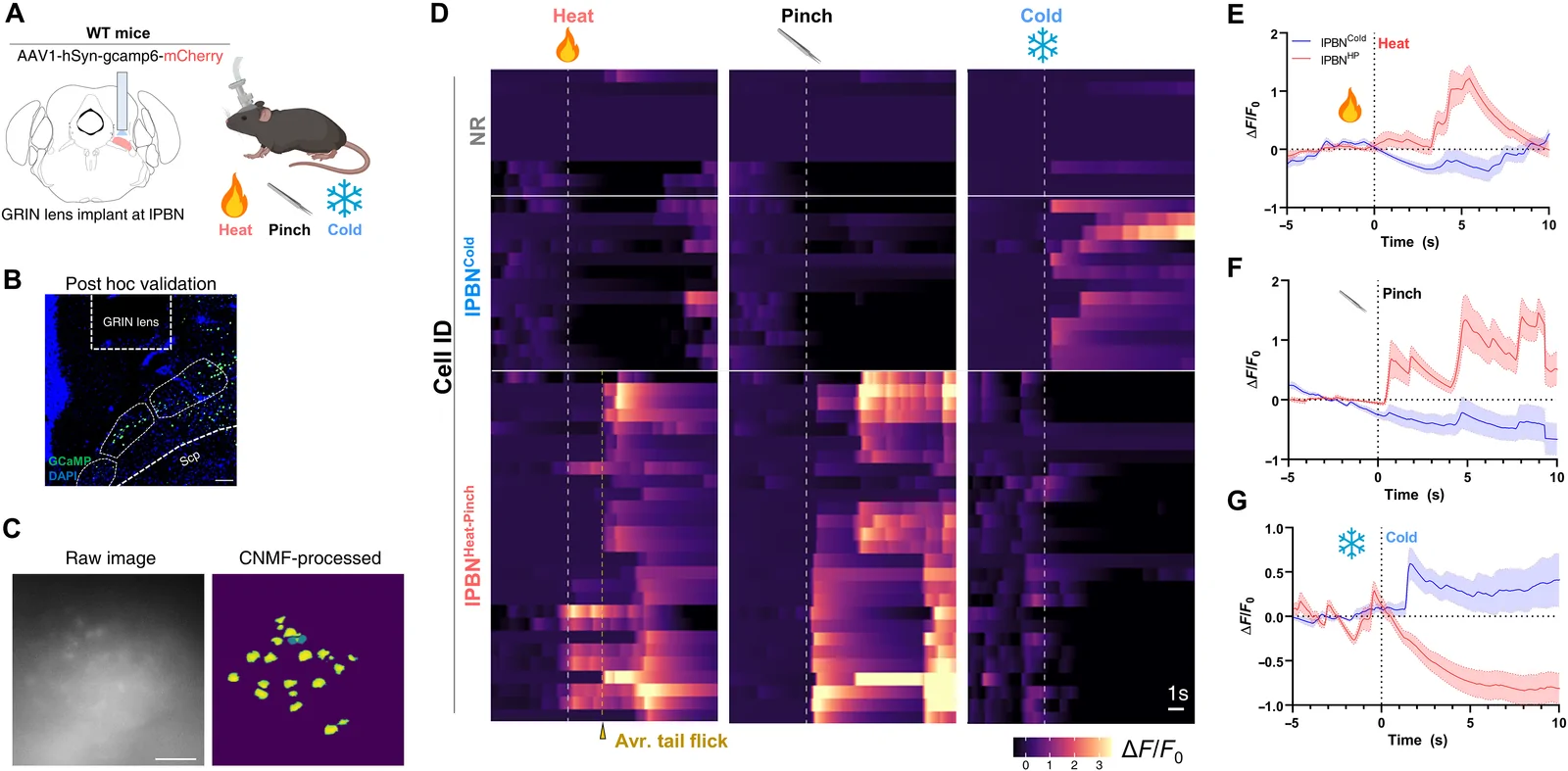
Response characteristics of lPBN neurons observed through in vivo calcium imaging. (**A**) Experimental scheme for the in vivo calcium imaging in lPBN. WT, wild type. Created in BioRender. Oh (2026), https://biorender.com/bgojiqn. (**B**) Examples of virus expression and location of GRIN lens implantation. Green: GCaMP6f; blue: DAPI. Scale bar, 100 μm. (**C**) Examples of single-cell imaging window in lPBN, both raw image from miniscope recording (left) and CNMF-processed image (right). Scale bar, 100 μm. (**D**) Heatmap representing activity of lPBN neurons during noxious heat, noxious pinching, and cold stimulation. The yellow dashed line marks the average nocifensive tail-flick latency of mice during noxious heat exposure. NR, nonresponsive neurons. (**E**) Peri-event plot of averaged fluorescent signal recorded in lPBN neurons in response to heat stimuli (red trace: hot-pinch–responsive neurons; blue trace: cold-responsive neurons). Responses of neurons are averaged by group and represented as the means ± SEM. (**F**) Peri-event plot of averaged fluorescent signal recorded in lPBN neurons in response to pinch stimuli (red trace: hot-pinch–responsive neurons; blue trace: cold-responsive neurons). Responses of neurons are averaged by group and represented as the means ± SEM. (**G**) Peri-event plot of averaged fluorescent signal recorded in lPBN neurons in response to cold stimuli (red trace: hot-pinch–responsive neurons; blue trace: cold-responsive neurons). Responses of neurons are averaged by group and represented as the means ± SEM.

### Chemogenetic activation of lPBN^cold^ neurons induces acute antinociception

To test the causal role of lPBN^cold^ neurons in analgesia, we first selectively activated lPBN^cold^ neurons using TRAP2 transgenic mice with the excitatory DREADD (designer receptor exclusively activated by designer drugs) hM3Dq (Fig. 3A). Using electrophysiological recordings in acute brain slices, we confirmed that treatment with clozapine-*N*-oxide (CNO) increased the activity of lPBN^cold^ neurons (Fig. 3, B and C, and fig. S5). Notably, we observed that 70.5% of recorded lPBN^cold^ neurons exhibited spontaneous activity (Fig. 3D), an observation that contrasts with previous reports suggesting that only a small subset of lPBN neurons exhibits spontaneous action potential firing (20 to 22% in both mice and rats) (*36*–*38*). CNO-induced activation of lPBN^cold^ neurons elicited both tonic (11 of 12 cells) and burst (1 of 12 cells) firing patterns (fig. S5D). However, the resting membrane potentials of quiescent cells and spontaneously active cells did not significantly differ (Fig. 3E). Activation of lPBN^cold^ neurons with intraperitoneal CNO injection increased the mechanical withdrawal threshold (Fig. 3F) and prolonged response latency to noxious heat (Fig. 3G) in a dose-dependent manner, while it decreased response latency and increased pain-like behavior scoring to noxious cold (Fig. 3, H and I). Nonetheless, CNO did not change any of these nocifensive behaviors in naïve mice, ruling out the nonspecific effects of CNO (1 mg/kg) (fig. S6). In addition to naïve states, intraperitoneal CNO injection was sufficient to reverse CFA-induced mechanical allodynia (Fig. 3J). The expression level of DREAAD in the bilateral lPBN correlated with CNO-induced antinociception, further supporting the role of lPBN neurons in antinociception (Fig. 3K). To further assess the behavioral effects of lPBN^cold^ neuron activation, we evaluated spontaneous exploration of a thermal gradient plate (fig. S7). Activation of lPBN^cold^ neurons reduced spontaneous entry into both noxious cold (below 17°C) and innocuous cold (between 17° and 24°C) zones in thermal gradient plates, indicating heightened cold sensitivity during lPBN^cold^ neuron activation (fig. S7, B to E). Nonetheless, activation of lPBN^cold^ neurons does not alter the core body temperature, excluding the thermoregulatory role of lPBN^cold^ neurons (fig. S7F).

**Fig. 3.**
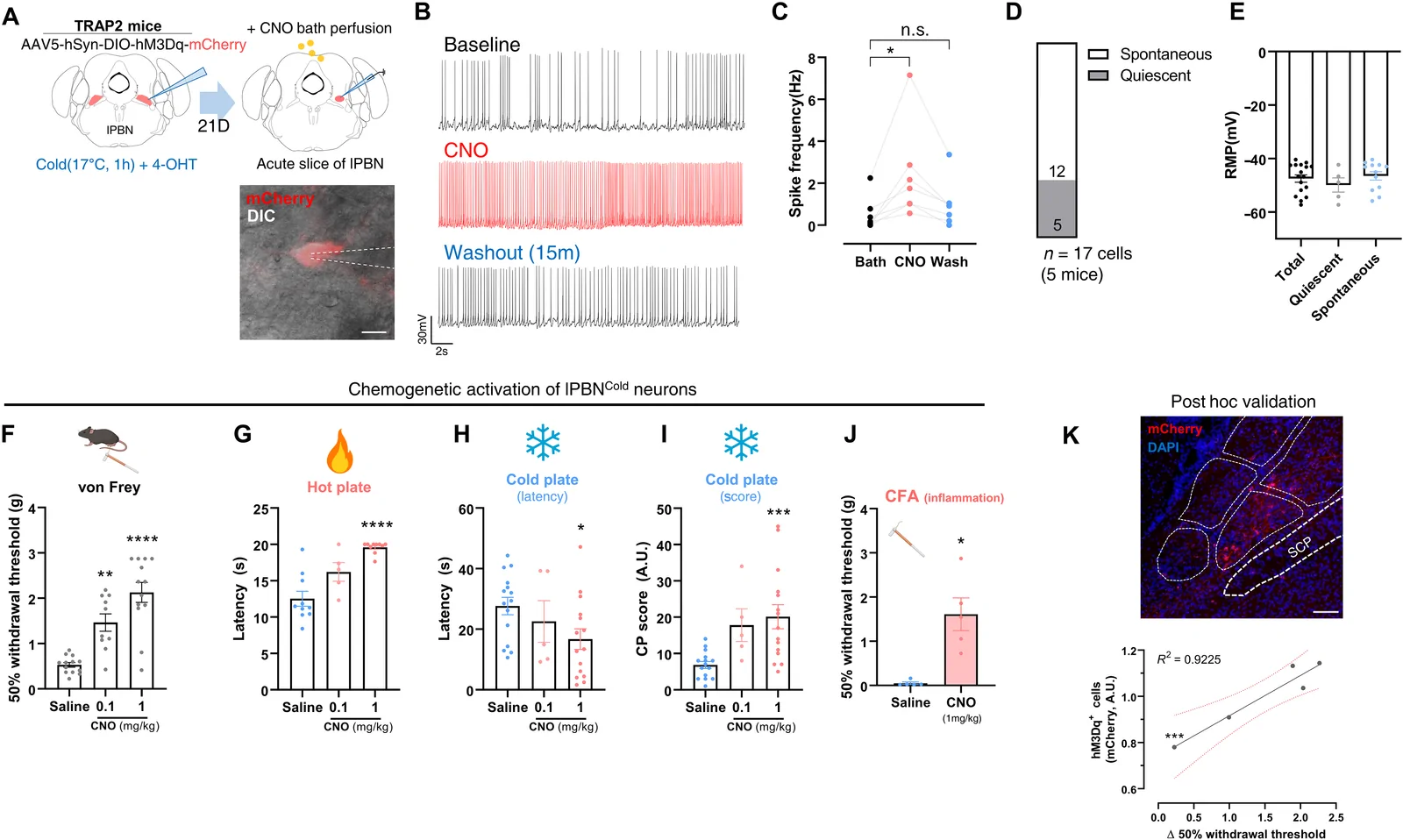
Chemogenetic activation of lPBN^cold^ neurons induces acute antinociception. (**A**) Experimental scheme for the experiments presented in (A) to (E). Red: mCherry. Scale bar, 10 μm. (**B**) Representative membrane potential trace of lPBN^cold^ neurons during CNO perfusion. (**C**) Quantification of CNO-induced spike frequency in lPBN^cold^ neurons. (**D**) Proportion of spontaneously firing neurons and quiescent neurons in recorded lPBN^cold^ neurons. (**E**) Resting membrane potential of recorded lPBN^cold^ neurons. (**F**) Mechanical sensitivity measured with 50% withdrawal thresholds in lPBN^cold^ neuron activation experiment. Created in BioRender. Oh (2026), https://biorender.com/6r5yeg9. (**G**) Thermal sensitivity to noxious heat measured with response latency to a hot plate in lPBN^cold^ neuron activation experiment. (**H**) Thermal sensitivity to noxious cold measured with response latency to a cold plate in lPBN^cold^ neuron activation experiment. (**I**) Thermal sensitivity to noxious cold measured with behavioral score to a cold plate in lPBN^cold^ neuron activation experiment. A.U., arbitrary units. (**J**) Mechanical sensitivity measured with 50% withdrawal thresholds in lPBN^cold^ neuron activation experiment, performed in CFA-induced inflammatory pain model mice. (**K**) Representative image of lPBN^cold^ neurons (top) and correlation plot of antinociceptive effect and expression of DREADD in lPBN (bottom). Red: mCherry; blue: DAPI. Scale bar, 100 μm. Data are presented as the means ± SEM. *P < 0.05, **P < 0.01, ***P < 0.001, and ****P < 0.0001. The number of mice and statistical tests are listed in table S1.

### Analgesic role of the lPBN^cold^-to-vlPAG neural circuit through opioid receptor signaling in vlPAG

To identify potential downstream targets of lPBN^cold^ neurons that mediate analgesic effects, we performed anterograde neural tracing on lPBN^cold^ neurons (Fig. 4A). Tracing experiments revealed axonal projection of lPBN^cold^ neurons to the ipsilateral PAG, mainly in the ventrolateral subcolumn, along with projections to paraventricular thalamus and medial hypothalamus (Fig. 4B). The involvement of vlPAG in pain relief is well established, and activation of lPBN to PAG projection induces antinociception according to a previous report (*24*). Moreover, we observed activation of vlPAG following cooling stimuli (Fig. 1E). Given that cold stimuli increased neuronal activity in the vlPAG and lPBN-vlPAG neural circuit has been previously implicated in antinociception (*25*), we sought to define the role of lPBN-vlPAG neural circuit in TRPM8-mediated analgesia. To functionally validate the role of vlPAG projection of the lPBN^cold^ neurons, we first investigated whether cold stimulation recruits the lPBN-to-vlPAG pathway. Cold stimuli induced Fos expression in 17.8% of vlPAG-projecting lPBN neurons, and 27.8% of cold-positive neurons were projecting to vlPAG (fig. S8; see table S1). Similar to global lPBN^cold^ neuron activation via intraperitoneal injection of CNO, selectively activating vlPAG projection of lPBN^cold^ neurons by CNO microinjection into bilateral vlPAG (Fig. 4C) also increased the mechanical and heat pain threshold while decreasing response latency to noxious cold (Fig. 4, D to G). To rule out potential confounds related to altered motivation, freezing behavior, or motor function, we conducted a sticky tape removal test applied to paw. Selective activation of the lPBN^cold^-vlPAG projection did not affect the latency to attending for the tape attached at forepaw nor the latency to remove the tape, indicating that the observed analgesic effects were not attributable to generalized suppression of motor function and/or reduced motivational drive to respond (Fig. 4H). Given that the vlPAG is a critical hub for endogenous opioid-mediated analgesia and cold-induced analgesia requires opioid receptor signaling (*39*, *40*), we examined whether local opioid receptor signaling in the vlPAG contributes to lPBN^cold^ neuron activation–induced analgesia. To test this hypothesis, we microinjected the opioid receptor antagonist naloxone into the bilateral vlPAG while activating the lPBN^cold^-vlPAG neural circuit. Local naloxone treatment into vlPAG attenuated the mechanical antinociception induced by lPBN^cold^-vlPAG circuit activation, suggesting that opioid receptor signaling in the vlPAG is necessary for lPBN^cold^-vlPAG neural circuit–mediated analgesia (Fig. 4I). Last, chemogenetic activation of lPBN^cold^-vlPAG neural circuit was sufficient to reverse CFA-induced mechanical allodynia (Fig. 4J). Collectively, our data demonstrated the antinociceptive role of lPBN^cold^-vlPAG neural circuit, which is mediated by local opioid receptor signaling in the vlPAG.

**Fig. 4.**
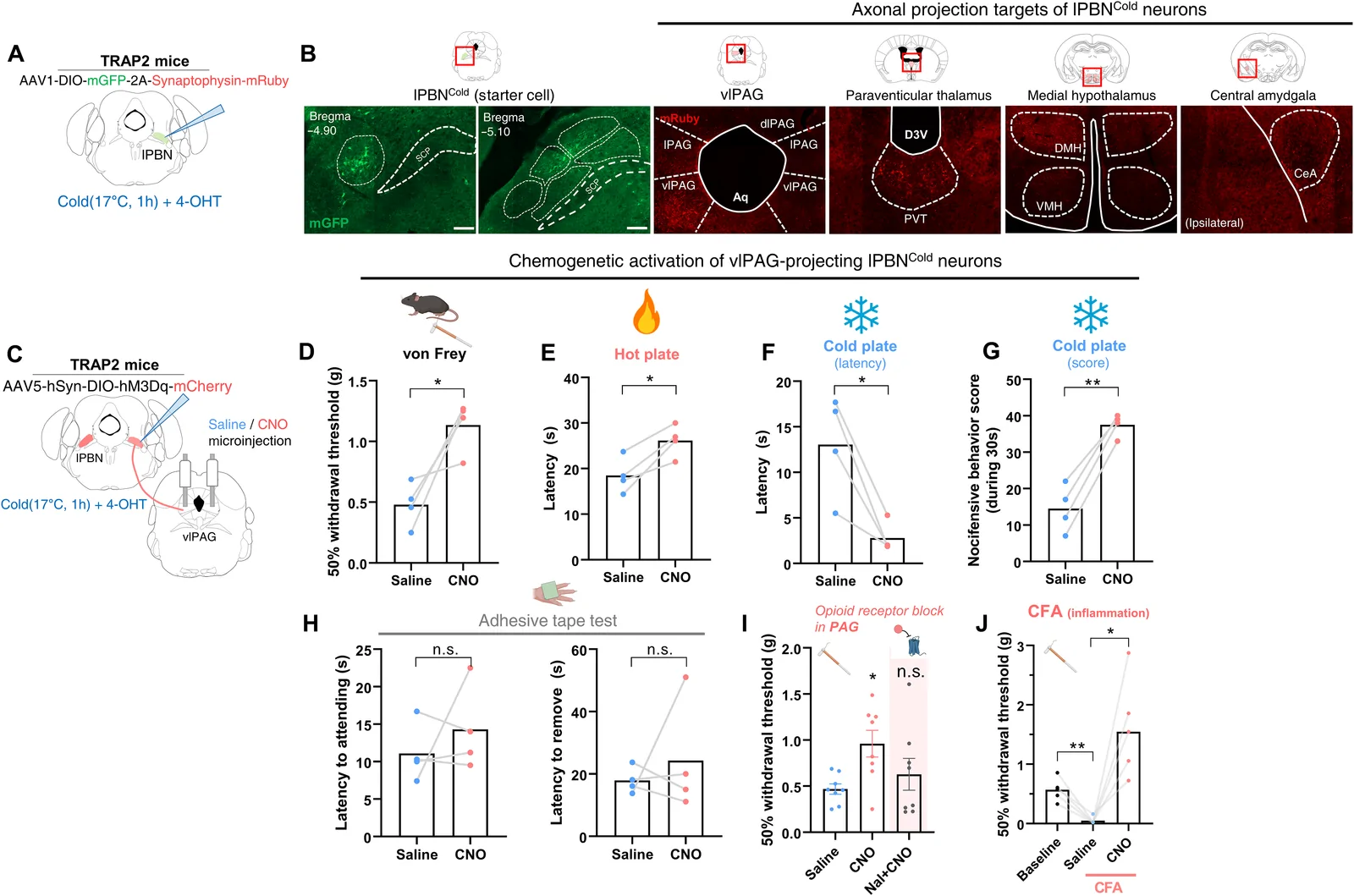
Analgesic role of the lPBN^cold^-to-vlPAG neural circuit through opioid receptor signaling in vlPAG. (**A**) Experimental scheme for the anterograde tracing of lPBN^cold^ neurons. (**B**) Distribution of GFP (green fluorescent protein)–positive neurons and neuronal axon projection in a diverse brain region. Green: GFP; red: synaptophysin-mRuby. Scale bars, 100 μm. (**C**) Experimental scheme for the selective activation of vlPAG-projecting lPBN^cold^ neurons. (**D**) Mechanical sensitivity measured with 50% withdrawal thresholds in vlPAG-projecting lPBN^cold^ neuron activation experiment. Created in BioRender. Oh (2026), https://biorender.com/6r5yeg9. (**E**) Thermal sensitivity to noxious heat measured with response latency to a hot plate in vlPAG-projecting lPBN^cold^ neuron activation experiment. (**F**) Thermal sensitivity to noxious cold measured with response latency to a cold plate in vlPAG-projecting lPBN^cold^ neuron activation experiment. (**G**) Thermal sensitivity to noxious cold measured with behavioral score to a cold plate in vlPAG-projecting lPBN^cold^ neuron activation experiment. (**H**) Latency to first attempt to tape removal in vlPAG-projecting lPBN^cold^ neuron activation experiment (left) and latency to tape removal in vlPAG-projecting lPBN^cold^ neuron activation experiment (right). Created in BioRender. Oh (2026), https://biorender.com/jclvvkx. (**I**) Mechanical sensitivity measured with 50% withdrawal thresholds in vlPAG-projecting lPBN^cold^ neuron activation experiment, followed by local naloxone injection. Created in BioRender. Oh (2026), https://biorender.com/jclvvkx. (**J**) Mechanical sensitivity measured with 50% withdrawal thresholds in vlPAG-projecting lPBN^cold^ neuron activation experiment, performed in CFA-induced inflammatory pain model mice. Data are presented as the means ± SEM. *P < 0.05 and **P < 0.01. The number of mice and statistical tests are listed in table S1.

### lPBN^cold^ neurons are necessary for TRPM8-dependent analgesia

Next, we tested whether lPBN^cold^ neurons and their downstream projections are necessary for TRPM8-dependent analgesia. To achieve this, we selectively ablated lPBN^cold^ neurons with Casp3 (Fig. 5, A to C). Given that previous studies have implicated the lPBN in homeostatic temperature regulation (*18*, *41*), we also evaluated the role of lPBN^cold^ neurons in thermoregulation and thermal preference, assessed by the preferred temperature in a thermal gradient test. Ablation of bilateral lPBN^cold^ neurons did not alter the baseline thermal preference and nociceptive sensitivity including mechanical and thermal pain (Fig. 5D and fig. S9, A to D and F to H). In addition, ablation of lPBN^cold^ neurons did not affect the core body temperature in the basal state measured with a rectal temperature probe (fig. S9E). To determine the necessity of lPBN^cold^ neurons for TRPM8-mediated analgesia, we used the same behavioral paradigm described in Fig. 1. To rule out the potential effects of icilin treatment procedure per se, which include light restrain and potential stress, we compared the mechanical withdrawal threshold between baseline (without any treatment) and vehicle-treated mice, which revealed no significant differences (Fig. 5E). Ablation of the lPBN^cold^ neurons attenuated mechanical antinociception (differences of 50% mechanical threshold, Δ*g*) induced by icilin administration to hindpaw in both naïve and CFA-induced inflammatory pain states (Fig. 5, F to H), demonstrating the essential role of lPBN^cold^ neurons in TRPM8-mediated analgesia. Furthermore, selective chemogenetic inactivation of the lPBN-to-vlPAG neural circuits phenocopied the loss of TRPM8-mediated analgesia, providing additional evidence that the lPBN-to-vlPAG circuit is a critical component of TRPM8-dependent pain modulation (Fig. 5, I and J).

**Fig. 5.**
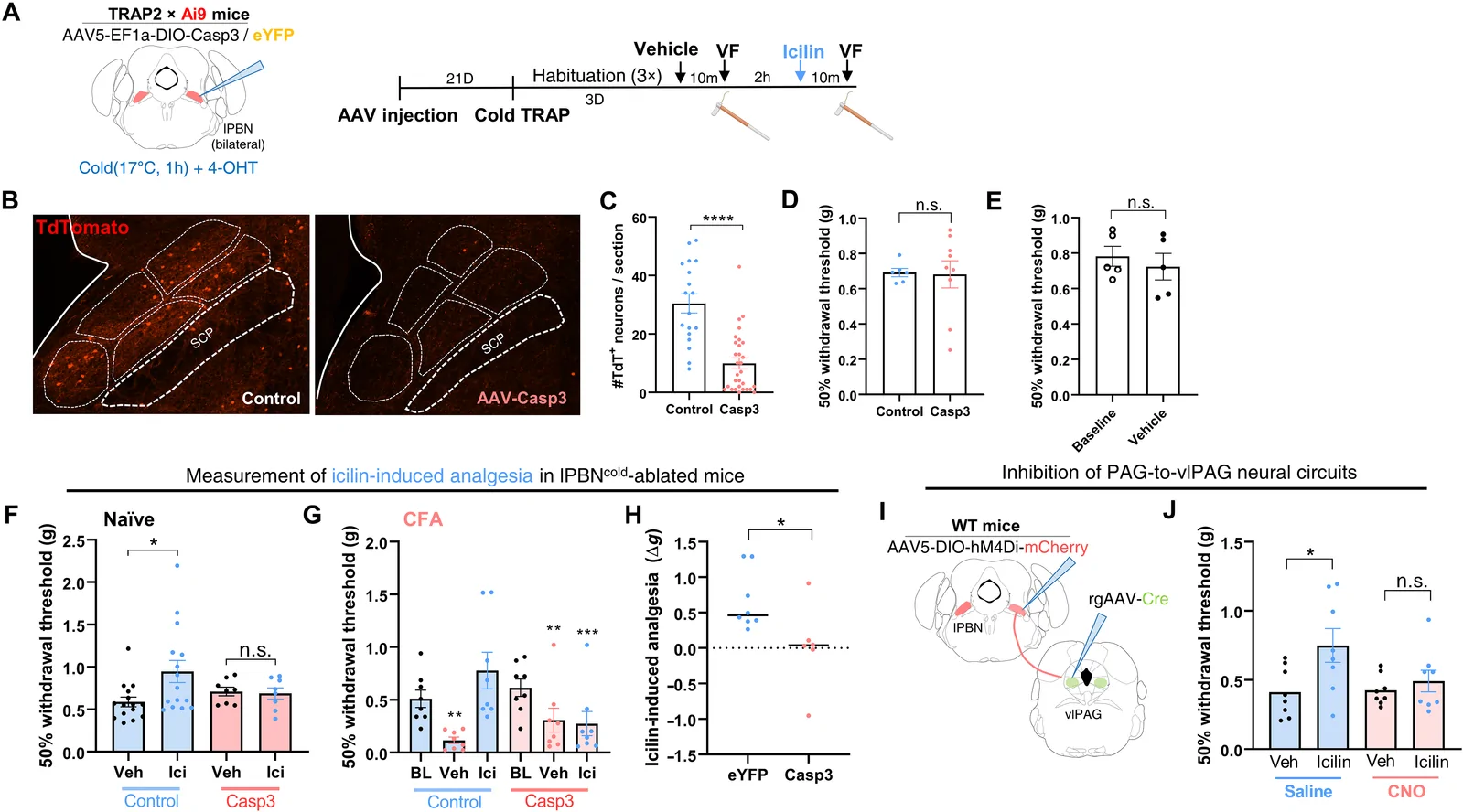
lPBN^cold^ neurons are necessary for TRPM8-dependent analgesia. (**A**) Experimental scheme and timeline for the icilin-induced pain relief experiment. eYFP, enhanced yellow fluorescent protein. (**B**) Representative images of lPBN in control and Casp3-mediated lPBN^cold^ neuron–ablated mice. Red: TdTomato. (**C**) Quantification of TdTomato^+^ neurons per lPBN section in control and Casp3-mediated lPBN^cold^ neuron–ablated mice. (**D**) Mechanical sensitivity measured with 50% withdrawal thresholds in control and Casp3-mediated lPBN^cold^ neuron–ablated mice. (**E**) Mechanical sensitivity measured with 50% withdrawal thresholds in control mice before and after vehicle treatment. (**F**) Mechanical sensitivity following icilin treatment in control mice and Casp3-mediated lPBN^cold^ neuron–ablated naïve mice. (**G**) Mechanical sensitivity following icilin treatment in control mice and Casp3-mediated lPBN^cold^ neuron–ablated mice after intraplantar CFA injection. (**H**) Quantification of topical icilin–induced antinociception (measured by difference of 50% withdrawal threshold) in control mice and Casp3-mediated lPBN^cold^ neuron–ablated mice. (**I**) Experimental scheme for lPBN-to-vlPAG neuronal circuit inhibition. (**J**) Mechanical sensitivity following icilin treatment in saline- and CNO-treated mice for lPBN-to-vlPAG neuron inhibition. Data are presented as the means ± SEM. *P < 0.05, **P < 0.01, ***P < 0.001, and ****P < 0.0001. The number of mice and statistical tests are listed in table S1.

### Identification of enkephalinergic signaling within vlPAG in TRPM8-dependent analgesia via transcriptomic profiling of lPBN^cold^ neurons

The lPBN is a highly heterogeneous structure, composed of multiple histologically and genetically distinct subnuclei, each with specialized physiological functions (*33*). To genetically characterize lPBN^cold^ neurons, we used RNA sequencing combined with fluorescence-activated cell sorting (FACS) (see Materials and Methods; Fig. 6A). The prepared single-cell suspension obtained from lPBN was sorted on the basis of green fluorescence (indicative of cold responsiveness) and 4′,6-diamidino-2-phenylindole (DAPI; indicates cell integrity and viability) through FACS, yielding 79.34% viable cells and 5.88% Zsg-positive cells among singlet gated counts (Fig. 6, B to D). To determine differentially expressed genes, we performed RNA sequencing on sorted viable Zsg-positive and Zsg-negative populations. Comparative analysis identified 207 significantly enriched genes and 1036 significantly depleted genes in Zsg-positive cells relative to Zsg-negative cells (Fig. 6E). As Zsg-positive, TRAP cells were predominantly neurons (*42*), classical markers for microglia (*Cx3cr1* and *Tmem119*), astrocyte (*Gfap* and *S100b*), and oligodendrocytes (*Mag* and *Mbp*) were significantly depleted or not enriched in Zsg-positive cells, but general housekeeping genes (*Actb* and *Gapdh*) remained unchanged between groups. Among the significantly enriched genes, we annotated genes associated with neuropeptide signaling, endogenous opioid pathways, and established marker genes for the parabrachial nucleus. Notably, *Penk* (proenkephalin), a precursor for enkephalin, was significantly enriched in Zsg-positive cells, and RNAscope fluorescence in situ hybridization analysis validated that 46.2% of cold-TRAP (Zsg-positive) lPBN neurons express RNA transcript for *Penk* (Fig. 6F). This suggests a potential molecular basis for cold-induced analgesia via endogenous opioid signaling. Chemogenetic activation of lPBN^cold^ neurons increased the punctate enkephalin immunoreactivity in the vlPAG, suggesting the functional expression, axonal transport, and/or presynaptic accumulation of enkephalin by lPBN^cold^ neuron activation (Fig. 6, G to I). Moreover, exposing hindpaws to cold increased the enkephalin immunoreactivity in vlPAG in naïve, wild-type (WT) mice (Fig. 6J). As previous reports demonstrated that enkephalin signaling within the vlPAG mediates an analgesic effect (*43*, *44*), and given the previous result suggesting that local opioid receptor signaling was necessary for lPBN^cold^-vlPAG neural circuit–mediated antinociception (Fig. 4I), we hypothesized that enkephalin release in the vlPAG represents a potential synaptic mechanism underlying TRPM8-mediated analgesia. To directly test this hypothesis, we used a single-vector system to deliver *Staphylococcus aureus* Cas9 (SaCas9) and CRISPR guide RNA for *Penk* (AAV-DJ/8-CMV-FLEX-SaCas9-U6-sgPenk-HA) to selectively disrupt the enkephalin gene in lPBN neurons (Fig. 6K) (*45*, *46*). CRISPR-mediated in vivo *Penk* knockout in total lPBN neurons and lPBN^cold^ neurons both attenuated the mechanical antinociception induced by icilin administration to hindpaw compared to wild-type controls (Fig. 6L). These findings demonstrated that enkephalinergic signaling within the vlPAG plays a crucial role in mediating TRPM8-mediated analgesia.

**Fig. 6.**
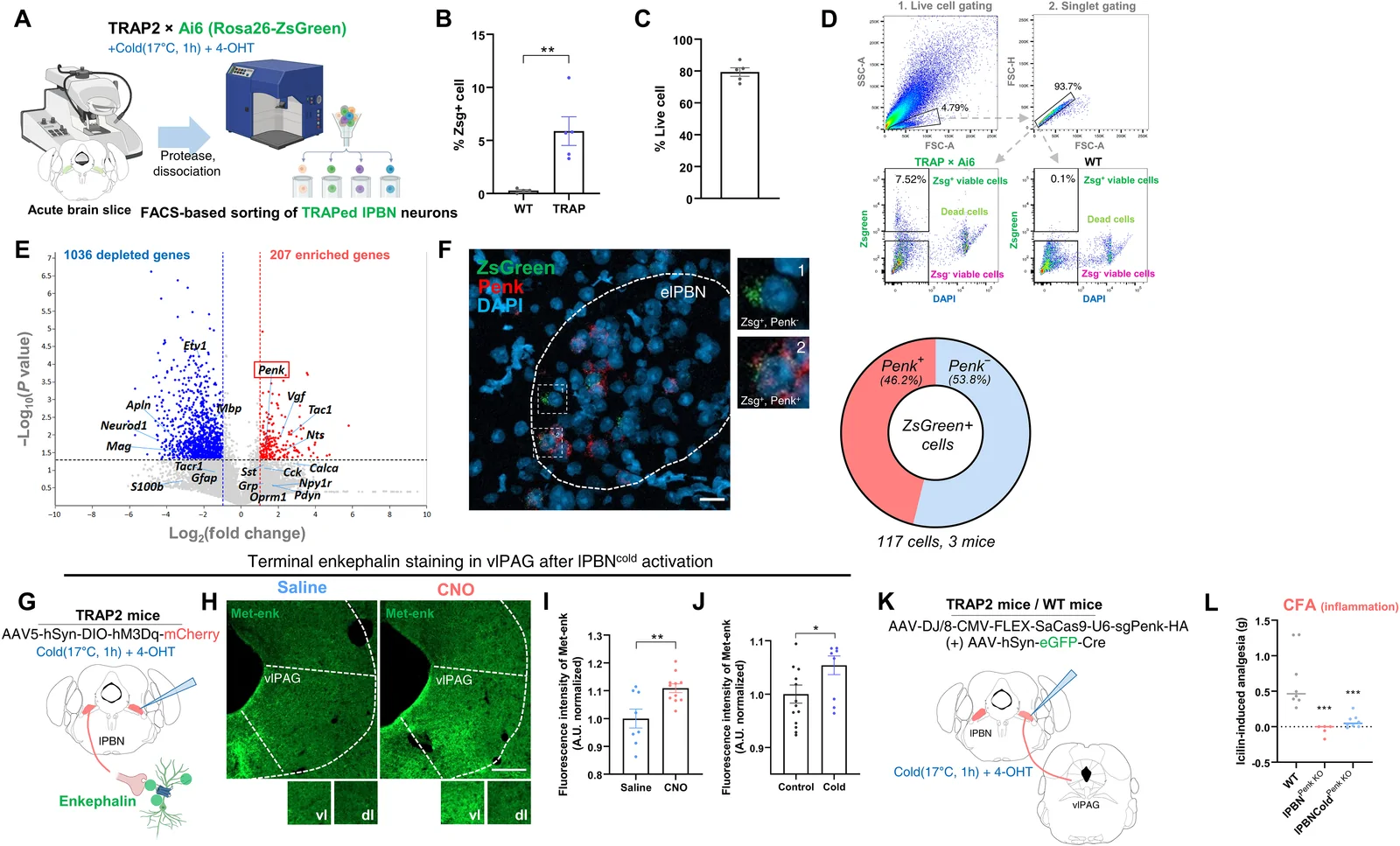
Identification of enkephalinergic signaling within vlPAG in TRPM8-dependent analgesia via transcriptomic profiling of lPBN^cold^ neurons. (**A**) Experimental scheme for preparation of single-cell suspension and sorting of Zsg-positive neurons of lPBN. Created in BioRender. Oh (2026), https://biorender.com/riy8hbg. (**B**) Percentage of Zsg-positive cells in total viable cells in wild-type mice and TRAP2 × Ai6 mice. (**C**) Percentage of viable cells in total counts. (**D**) Gating strategy for fluorescence-based sorting of Zsg-positive, viable neurons from brain single-cell suspension. (**E**) Volcano plot of significantly enriched genes (red dot) and depleted genes (blue dot) in Zsg-positive cells compared to Zsg-negative cells. (**F**) Representative figure of RNAscope fluorescence in situ hybridization in lPBN (left) and quantification of marker coexpression in Zsg-expressing lPBN neurons (right). Red: Penk; green: ZsGreen; blue: DAPI (left). Scale bar, 20 μm. (**G**) Experimental scheme for the selective activation of lPBN^cold^ neurons for enkephalin immunostaining. (**H**) Representative figure of met-enkephalin immunostaining in vlPAG with lPBN^cold^ neuron activity manipulation (top) and enlarged images from ventrolateral subcolumn (vl) and dorsolateral subcolumn (dl) (bottom). Green: met-enkephalin. Scale bar, 100 μm. (**I**) Quantification of met-enkephalin immunoreactivity in vlPAG after lPBN^cold^ neuron activity manipulation. (**J**) Quantification of met-enkephalin immunoreactivity in vlPAG, in control mice and cold-exposed mice. (**K**) Experimental scheme for the neuron-specific *Penk* knockout in lPBN neurons before TRPM8-mediated analgesia behavioral tests. (**L**) Mechanical sensitivity measured with 50% withdrawal thresholds in the CFA-induced inflammatory pain model, with in vivo *Penk* knockout in lPBN neuron populations. Data are presented as the means ± SEM. *P < 0.05, **P < 0.01, and ***P < 0.001. The number of mice and statistical tests are listed in table S1.

### High-frequency activation of lPBN^cold^ neurons engages RVM-projecting vlPAG neurons through enkephalin-dependent disinhibition

The vlPAG contains opioid receptor–expressing neurons that play a crucial role in endogenous opioid-mediated analgesia and descending pain modulation through their interactions with other brainstem regions such as the locus coeruleus and the rostral ventromedial medulla (RVM) (*47*, *48*). These structures are known to project to the spinal cord and induce antinociception via noradrenergic or serotonergic signaling, respectively (*49*). Thus, we investigated whether lPBN^cold^ neurons functionally engage these pathways to mediate the analgesic effect. First, we performed double labeling combined with ex vivo electrophysiology (Fig. 7A). We expressed channelrhodopsin-2 (ChR2) in lPBN^cold^ neurons using TRAP mice and then labeled RVM-projecting vlPAG neurons (which are key components of the descending pain modulation system) through injection of the retrograde tracer cholera toxin B conjugated with Alexa Fluor 488 (CTB-488) in the RVM (*48*). Whole-cell patch-clamp recordings showed that optogenetic blue light stimulation consistently evoked action potentials in lPBN^cold^ neurons in a time-locked manner (Fig. 7B). To determine how activation of the lPBN^cold^ neuron terminal affects the vlPAG-RVM circuit, we monitored the postsynaptic events in RVM-projecting CTB-488^+^ vlPAG neurons (vlPAG^CTB+^ neurons) during optogenetic lPBN^cold^ neuron axon terminal activation. Although both excitatory postsynaptic currents (EPSCs) and inhibitory postsynaptic currents (IPSCs) were observed, only IPSCs were time locked to optogenetic stimulation, suggesting an inhibitory effect of lPBN^cold^ neuron activation on vlPAG^CTB+^ neurons (Fig. 7C). Next, to characterize the underlying synaptic architecture, we performed Gaussian fitting of optogenetically induced IPSCs (oIPSCs) to calculate the mean latency and synaptic jitter. The mean latency was 11.02 ms, and the synaptic jitter was 1.24 ms, consistent with a polysynaptic, potentially disynaptic, feedforward inhibitory response (*50*). Given the predominantly glutamatergic nature of lPBN neurons, these findings suggest the recruitment of a local feedforward inhibitory microcircuit in the vlPAG (Fig. 7D). Thus, we hypothesized that high-frequency stimulation, which is generally more favorable for neuropeptide release from large dense-core vesicles than low-frequency stimulation (*51*, *52*), could recruit enkephalin-dependent opioid signaling and suppress feedforward inhibition, thereby depolarizing vlPAG^CTB+^ neurons. We selected 5 Hz as a low-frequency comparison condition and 40 Hz as a high-frequency stimulation condition on the basis of a previous study demonstrating the preferential recruitment of dense-core vesicles by higher-frequency neuronal activity in the lPBN neurons (*53*). 40-Hz, but not 5-Hz, stimulation produced prolonged depolarization of vlPAG^CTB+^ neurons, with a mean depolarization latency of 7.05 s after stimulation (Fig. 7E). Moreover, this depolarizing effect was blocked by naloxone pretreatment (Fig. 7, E and F), suggesting that high-frequency stimulation of lPBN^cold^ neuron terminals depolarizes vlPAG-RVM projection neurons in an opioid receptor–dependent manner. Consistent with this model, 40-Hz optogenetic stimulation decreased the frequency of IPSCs in vlPAG^CTB+^ neurons, an effect that was blocked by naloxone (Fig. 7G). Last, to directly test the role of enkephalin in lPBN^cold^ neurons, we repeated the same experiment after co-delivering SaCas9 and sgPenk constructs to lPBN^cold^ neurons, thereby knocking out *Penk* in lPBN^cold^ neurons (Fig. 7H and fig. S10). As expected, disruption of the *Penk* gene in lPBN^cold^ neurons abolished high-frequency stimulation–induced neuronal depolarization in vlPAG^CTB+^ neurons compared to wild-type controls (Fig. 7, I to K). Together, these electrophysiological findings support a model in which high-frequency activation of lPBN^cold^ inputs recruits enkephalin-dependent opioid signaling that disinhibits RVM-projecting vlPAG neurons. Although these results do not exclude concurrent glutamatergic cotransmission from lPBN^cold^ neurons, they support a critical role for enkephalin-dependent signaling in the prolonged depolarization of vlPAG^CTB+^ neurons observed under our high-frequency stimulation.

**Fig. 7.**
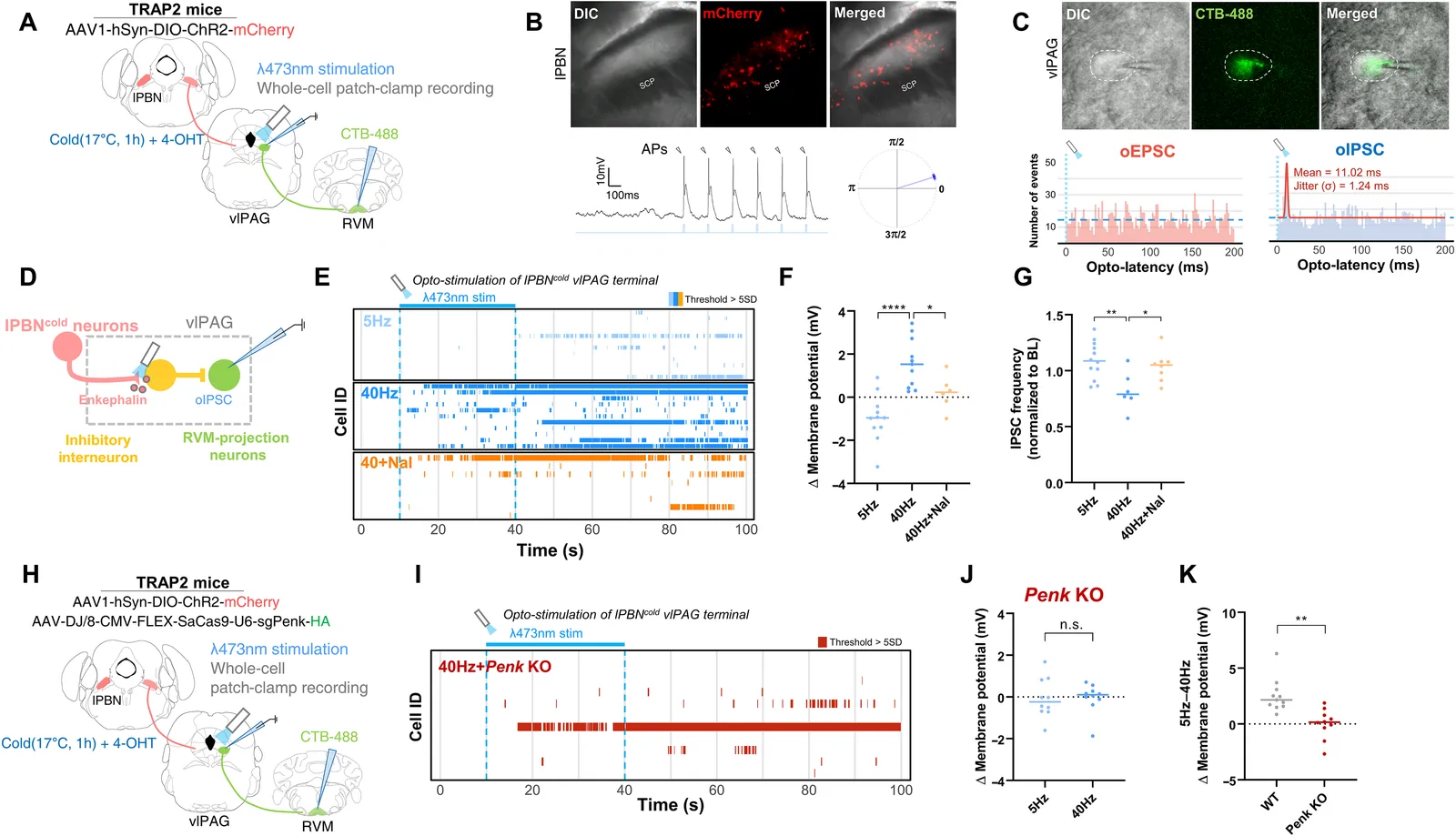
High-frequency activation of lPBN^cold^ neurons engages RVM-projecting vlPAG neurons through enkephalin-dependent disinhibition. (**A**) Experimental scheme for eletrophysiological recording in vlPAG^CTB+^ neurons during lPBN^cold^ neuron stimulation. (**B**) Representative image of ChR2-mCherry–expressing lPBN^cold^ neurons. Red: mCherry (top). Representative membrane voltage trace showing action potential (AP) firing during optogenetic stimulation (bottom left) and phase diagram showing the relative phase of optogenetic stimulation and action potential firing (bottom right). (**C**) Representative image of CTB488-expressing vlPAG^CTB+^ neuron. Green: CTB-488 (top). Plots for optogenetic latency analysis of EPSCs and IPSCs. Baseline-level currents are denoted in blue, and dashed lines and Gaussian curve fitting of oIPSC are shown in red lines (bottom). (**D**) Schematic diagram of putative microcircuits in vlPAG. (**E**) Heatmaps showing the averaged membrane potential of vlPAG^CTB+^ neurons during optogenetic stimulation of the lPBN^cold^ neuronal terminal. Only increases in membrane potential exceeding 5 standard deviations above the baseline are shown. (**F**) Quantification of vlPAG^CTB+^ neuron potential change during optogenetic stimulation of the lPBN^cold^ neuron terminal. (**G**) Quantification of IPSC frequency in vlPAG^CTB+^ neurons during optogenetic stimulation of the lPBN^cold^ neuron terminal. BL, baseline. (**H**) Experimental scheme for eletrophysiological recording in vlPAG^CTB+^ neurons during lPBN^cold/Penk KO^ neuron stimulation. (**I**) Heatmaps showing the average membrane potential of vlPAG^CTB+^ neurons during 40-Hz optogenetic stimulation in the brain slice of conditional *Penk* KO mice. Only increases in membrane potential exceeding 5 standard deviations above the baseline are shown. (**J**) Quantification of vlPAG^CTB+^ neuron potential change during optogenetic stimulation of the lPBN^cold/Penk KO^ neuron terminal. (**K**) Comparison of membrane potential change during optogenetic stimulation of the lPBN^cold/Penk KO^ neuron terminal in wild-type and *Penk* cKO mice. Data are presented as the means. *P < 0.05, **P < 0.01, ***P < 0.001, and ****P < 0.0001. The number of mice and statistical tests are listed in table S1.

### Activation of lPBN^cold^ neurons decreases Fos expression to noxious stimuli in the spinal cord, with involvement of the descending pain modulation circuit

As demonstrated by the eletrophysiological recording, activation of lPBN^cold^ neurons engages the vlPAG-RVM neuronal circuit through released enkephalin and opioid receptor–mediated disinhibition (Fig. 7). Considering the well-established role of vlPAG-RVM neuronal circuit in descending pain modulation, we hypothesized that activation of lPBN^cold^ neurons reduces nocifensive responses through descending pain modulation from the brainstem to the spinal cord. To functionally assess the role of lPBN^cold^ neurons in descending pain modulation, we chemogenetically activated lPBN^cold^ neurons and repeatedly applied a noxious heat stimulus (55°C) to both hindpaws. After 90 min, Fos expression induced by noxious heat stimulation was assessed in the lumbar spinal cord (L3 to L5) (Fig. 8A). As expected, activation of lPBN^cold^ neurons reduced noxious heat–induced Fos in both inhibitory and excitatory neurons within the superficial layer of the spinal cord [a primary synapse point for nociceptive sensory neurons, which was identified by isolectin-B4 (IB4) staining], with a greater proportional reduction observed in excitatory neurons than in inhibitory neurons (Fig. 8, B to D). Moreover, a specific subset of neurons in the superficial layers transmits nociceptive signals to the brain (spinal projection neuron). To determine whether chemogenetic activation of lPBN^cold^ neurons also decreased the activity of these projection neurons, we injected CTB-488 into the lPBN to visualize spinoparabrachial projection neurons (SPB neurons), which are crucial for nocifensive behavior and aversive learning (*54*). Chemogenetic activation of lPBN^cold^ neurons decreased noxious heat–induced Fos expression in SPB neurons (Fig. 8, E and F). Moreover, to assess the broader population of lamina I projection neurons, including but not limited to SPB neurons, we costained for neurokinin 1 receptor (NK1R), a canonical marker of spinal cord projection neurons (*34*, *55*). Similarly, we observed a decrease in noxious heat–induced Fos expression in NK1R^+^ spinal projection neurons (Fig. 8G). In the deep layers of the spinal cord, chemogenetic activation of lPBN^cold^ neurons reduced Fos expression specifically in excitatory neurons (Fig. 8, H to J). These results indicate that lPBN^cold^ neurons diminish spinal nociceptive output through descending pain modulation.

**Fig. 8.**
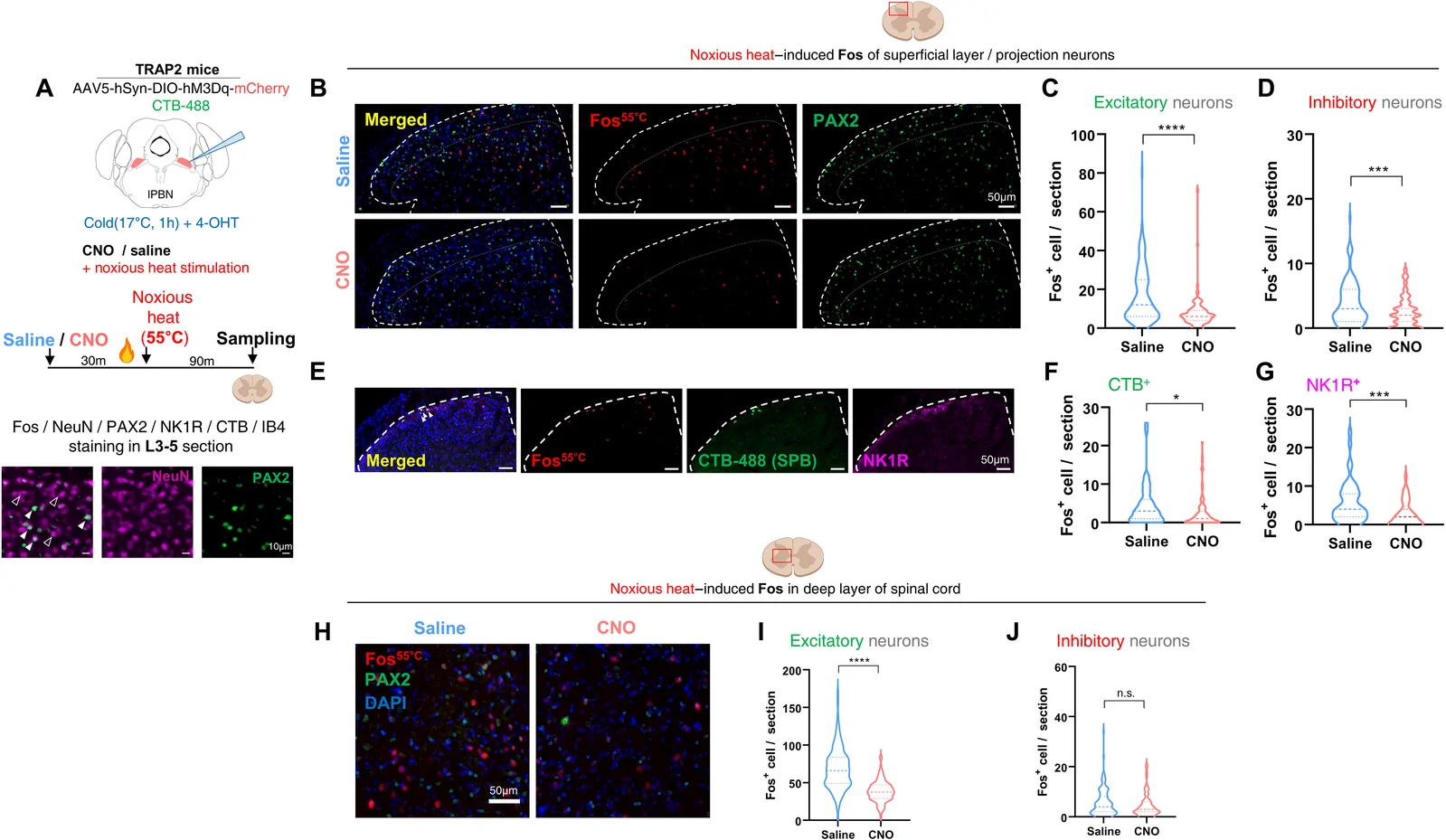
Activation of lPBN^cold^ neurons decreases Fos expression to noxious stimuli in the spinal cord, with involvement of the descending pain modulation circuit. (**A**) Experimental scheme for the experimental test presented here (top) and representative image of excitatory (open arrowhead) and inhibitory (solid arrowhead) neurons in the spinal cord. Green: PAX2; magenta: NeuN. Scale bars, 10 μm (bottom). (**B**) Distribution of Fos immunostaining in a superficial layer of lumbar 3-to-5 spinal cord in saline- and CNO-treated mice. Blue: DAPI; green: PAX2; red: noxious heat–induced Fos. Scale bars, 50 μm. (**C**) Quantification of Fos^+^ excitatory neurons in a superficial layer of lumbar 3-to-5 spinal cord in saline- and CNO-treated mice. (**D**) Quantification of Fos^+^ inhibitory neurons in superficial layer of lumbar 3-to-5 spinal cord in saline- and CNO-treated mice. (**E**) Distribution of Fos immunostaining in spinal projection neuron subsets. Blue: DAPI; green: CTB-488; red: noxious heat–induced Fos; violet: NK1R. Scale bars, 50 μm. (**F**) Quantification of Fos^+^, CTB^+^ neurons in a superficial layer of lumbar 3-to-5 spinal cord in saline- and CNO-treated mice. (**G**) Quantification of Fos^+^, NK1R^+^ neurons in a superficial layer of lumbar 3-to-5 spinal cord in saline- and CNO-treated mice. (**H**) Distribution of Fos immunostaining in a deep layer of lumbar 3-to-5 spinal cord in saline- and CNO-treated mice. Blue: DAPI; green: PAX2; red: noxious heat–induced Fos. Scale bar, 50 μm. (**I**) Quantification of Fos^+^ excitatory neurons in a deep layer of lumbar 3-to-5 spinal cord in saline- and CNO-treated mice. (**J**) Quantification of Fos^+^ inhibitory neurons in a deep layer of lumbar 3-to-5 spinal cord in saline- and CNO-treated mice. Data are presented as the means ± SEM. *P < 0.05, ***P < 0.001, and ****P < 0.0001. The number of mice and statistical tests are listed in table S1.

### Activation of the cold-activated descending pain modulation circuit decreases spinal responsiveness to noxious stimuli

Previous studies have shown that neurons reside in RVM, which contains several types of spinally projecting neurons, categorized on the basis of their responses to noxious stimuli: “on-cells,” “off-cells,” and “neutral cells” (*56*). On-cells are activated by noxious stimulation and are associated with pain facilitation, whereas off-cells are inhibited immediately before nociceptive reflexes and are involved in pain inhibition. To account for this functional heterogeneity and directly test the involvement of descending pain modulation mediated by pain-inhibitory RVM neurons, we selectively activated cold-responsive RVM neurons (RVM^cold^ neurons) by expressing the excitatory DREADD hM3Dq in RVM^cold^ neurons using TRAP2 transgenic mice (Fig. 9, A and B). Chemogenetic activation of RVM^cold^ neurons induced an antinociceptive effect to noxious mechanical and heat stimuli but not to noxious cold stimuli (Fig. 9, C to E), demonstrating modality-specific modulation of nociception. Consistent with these behavioral effects, chemogenetic activation of RVM^cold^ neurons decreased noxious heat–induced Fos expression in lumbar spinal cord neurons, including neurons in the superficial layers, spinal projection neurons (NK1R^+^), and neurons in the deep layers (Fig. 9, F to J). Collectively, these results suggest the involvement of lPBN^cold^ neurons in the descending pain modulation through antinociceptive RVM^cold^ neurons, further revealing the neural circuit mechanisms underlying cold-induced analgesia.

**Fig. 9.**
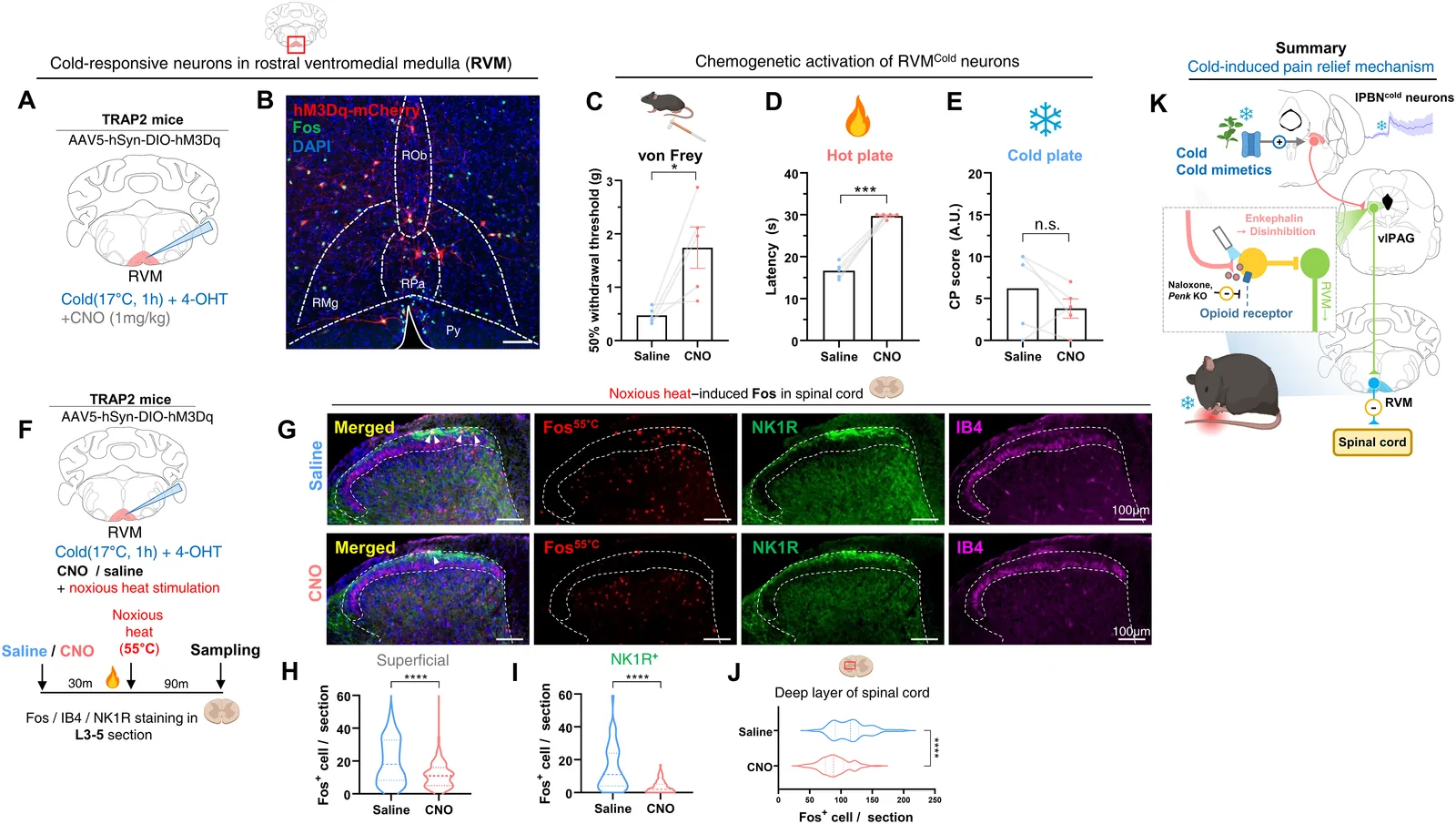
Activation of the cold-activated descending pain modulation circuit decreases spinal responsiveness to noxious stimuli. (**A**) Experimental scheme for the experimental test presented in (A) to (E). (**B**) Representative image of cold-TRAP RVM neurons. Blue: DAPI; green: Fos; red: mCherry. Scale bar, 100 μm. Rob, raphe obscurus nucleus; RMg, raphe magnus nucleus; RPal, raphe pallidus nucleus; Py, pyramidal tract. (**C**) Mechanical sensitivity measured with 50% withdrawal thresholds in RVM^cold^ neuron activation experiment. Created in BioRender. Oh (2026), https://biorender.com/6r5yeg9. (**D**) Thermal sensitivity to noxious heat measured with response latency to a hot plate in RVM^cold^ neuron activation experiment. (**E**) Thermal sensitivity to noxious cold measured with nocifensive behavioral score to a cold plate in RVM^cold^ neuron activation experiment. (**F**) Experimental scheme for the experimental test presented in (G) to (J). (**G**) Distribution of Fos immunostaining in the spinal superficial layer. Blue: DAPI; green: NK1R; red: noxious heat–induced Fos; violet: IB4. Scale bars, 50 μm. (**H**) Quantification of Fos^+^ cells in a superficial layer of lumbar 3-to-5 spinal cord in saline- and CNO-treated mice. (**I**) Quantification of Fos^+^, NK1R^+^ neurons in a superficial layer of lumbar 3-to-5 spinal cord in saline- and CNO-treated mice. (**J**) Quantification of Fos^+^ cells in a deep layer of lumbar 3-to-5 spinal cord in saline- and CNO-treated mice. (**K**) Schematic diagram for the suggested mechanism of cold-induced pain relief. Created in BioRender. Oh (2026), https://biorender.com/ae3a76f. Data are presented as the means ± SEM. *P < 0.05, ***P < 0.001, and ****P < 0.0001. The number of mice and statistical tests are listed in table S1.

## DISCUSSION

In this study, we first demonstrated that cold-responsive neurons within the lPBN govern TRPM8-dependent analgesia through projections to vlPAG by using viral tracing and chemogenetic activity modulation. In contrast, lPBN^cold^ neurons were dispensable for thermal preference and body temperature regulation. Moreover, through transcriptomic analysis combined with the TRAP method, we found that cold-responsive lPBN neurons were enriched with *Penk*, and TRPM8-dependent pain relief is mediated by local enkephalinergic signaling in the vlPAG. Last, activation of cold-responsive lPBN neurons diminished spinal responsiveness to noxious stimuli potentially through cold-responsive RVM neurons, revealing a cellular and molecular circuit mechanism contributing to cold-induced pain relief (Fig. 9K).

The lPBN comprises functionally divergent neuronal populations that process a wide range of sensory stimuli. While the previous literature reported that a distinct subpopulation of lPBN neurons is responsive to diverse sensory stimuli such as pruritic (itch-inducing) stimuli, painful pinch and noxious heat, temperature change, gustatory stimuli, and mechanical distension from the stomach (*17*, *57*–*61*), direct single-cell resolution observation of lPBN neuronal response to peripheral sensory modality has been limited. In this study, using in vivo calcium imaging, we identified distinct neuronal populations within the lPBN such that neurons responding to cold are separated from neurons responsive to noxious heat and pinch (Fig. 2). This observation aligns with prior extracellular unit recordings in lPBN, which identified cold-sensitive neurons, but not those responsive to noxious heat and mechanical pinch stimuli, without somatotopic arrangement (*35*). In addition, in vivo calcium imaging of SPB neurons in the spinal dorsal horn and whole-cell patch-clamp recording of SPB neurons with skin-nerve preparation showed the similar responsive pattern, in which a subset of SPB neurons is selectively activated by cold but not by mechanical indentation and noxious heat (*62*, *63*). Given that SPB neurons relay primary sensory input from the periphery to lPBN, it is likely that the sensory responsiveness pattern of lPBN neurons is largely driven by SPB neurons.

Previous studies have identified cold-responsive neurons within the lPBN primarily in the context of a homeostatic thermoregulation and thermal preference, as activation of dorsomedial hypothalamic nucleus (DMH)–projecting lPBN neurons induced thermogenesis, and ablation of lPBN impaired the normal thermal preference (*17*, *18*, *41*). In contrast, in the present study, neither activation nor inactivation of lPBN^cold^ neurons altered the core body temperature (figs. S7F and S9E), and ablation of lPBN^cold^ neurons did not change the thermal preference (fig. S9). Instead, activation of lPBN^cold^ neurons modulates acute nociceptive responsiveness (Fig. 3, F to I), and anterograde tracing of lPBN^cold^ neurons showed relatively sparse DMH projection (Fig. 4B). Moreover, RNA sequencing analysis showed lack of somatostatin enrichment in lPBN^cold^ neurons (Fig. 6E). This physiological, anatomical, transcriptomic, and behavioral evidence suggests that the lPBN^cold^ neuronal population identified in the current study is distinct from DMH-projecting, somatostatin-positive lPBN neurons, which are implicated in thermoregulation in a previous report (*18*). This discrepancy may stem from differences in the stimuli used to label cold-responsive neurons. In our study, cold-responsive neurons were identified and manipulated following relatively short exposure to innocuous cold stimulation with normal ambient temperature, primarily targeting the paw and tail regions via placement on the metal plate (Fig. 1 and fig. S1; see Materials and Methods), while aforementioned studies exposed the mouse to the cold chamber for a longer time period, which would challenge and compromise the core body temperature (*17*, *18*). Accordingly, it is plausible that neurons responsive to core body temperature change that governs long-term, homeostatic thermoregulation are functionally distinguished from those involved in short-term, acute behavioral response regulation to sensory stimuli in the lPBN. Nonetheless, the precise mechanisms by which these neural populations encode different sensory stimuli and regulate distinct physiological functions require further investigation.

In the present study, we identified a subset of lPBN neurons projecting to the vlPAG that exert antinociceptive effects (Fig. 4, D and E). Notably, the axonal projection of lPBN^cold^ neurons was predominantly localized in the vlPAG, whereas prior tracing studies have shown that noxious stimulus–responsive TacR1^+^ lPBN neurons primarily target the dorsolateral division of PAG, a region implicated in active flight and nocifensive response (*33*, *58*). Furthermore, we found that most of these cold-responsive lPBN neurons are *Penk*^+^ neurons, suggesting that they constitute a previously uncharacterized subpopulation of lPBN neurons that mediates analgesic actions through enkephalin-dependent opioid signaling in the vlPAG (Figs. 6, G to L, and 7). The vlPAG is one of the most well-characterized brain regions involved in opioid-mediated analgesia, including enkephalin-mediated analgesia (*44*, *64*–*67*). For instance, previous studies have demonstrated that microinjection of enkephalin or enkephalinase inhibitor into vlPAG is antinociceptive (*68*, *69*). Moreover, administration of an anti-enkephalin neutralizing antibody into vlPAG diminishes the analgesic effect of electroacupuncture, highlighting the functional role of enkephalin signaling in vlPAG in pain suppression (*43*). To note, conventional tracing and anatomical studies have confirmed the presence of enkephalinergic neuronal terminals in vlPAG, yet the precise source of enkephalinergic neuronal terminals has remained elusive (*70*). Recent evidence has shown that the lPBN contains enkephalinergic neurons that project to the vlPAG (*44*), which aligns with current experimental results.

Moreover, our study revealed that activation of lPBN^cold^ neurons and cold exposure increased enkephalin immunoreactivity in the vlPAG (Fig. 6, I and J), consistent with enhanced recruitment of enkephalinergic signaling in this region. Together with the naloxone-sensitive depolarization of RVM-projecting vlPAG neurons and the loss of this response following *Penk* disruption in lPBN^cold^ neurons, these findings support a model in which lPBN^cold^-derived *Penk*-dependent opioid signaling contributes to the activation of the descending pain-modulatory circuit. In particular, most of lPBN^cold^ neurons displayed spontaneous activity (Fig. 3, B and C, and fig. S5), raising the possibility that these neurons may contribute to tonic opioid peptide signaling in the PAG (*71*). Previous studies have consistently demonstrated that cold-induced analgesia relies on opioid receptor signaling, but the direct action of this signaling has remained elusive (*1*, *4*, *13*). Bridging a knowledge gap, we first demonstrated that lPBN-derived enkephalinergic signaling in the vlPAG contributes to TRPM8-induced analgesia (Figs. 6L and 7) via the RVM-mediated descending pain modulation pathway (Figs. 7 to 9). Supporting our experimental data, neuroimaging studies conducted in human subjects have demonstrated that analgesic cold stimuli activate the PAG (*49*, *72*, *73*), and pain relief induced by skin cooling and menthol is attenuated by the opioid receptor antagonist naloxone (*1*, *4*, *13*). This suggested that cold-induced analgesia could also be mediated in humans through the central pathway involving the PAG, enkephalinergic opioid receptor signaling, and the descending pain inhibitory circuit.

An important limitation of the present study is that our experiments do not directly visualize enkephalin release from lPBN^cold^ terminals or define its temporal dynamics in vivo. Although the increased enkephalin immunoreactivity, naloxone sensitivity, and *Penk*-specific loss-of-function experiments collectively support the involvement of enkephalinergic signaling, these approaches cannot directly resolve peptide release kinetics in the vlPAG. Future studies using genetically encoded opioid peptide sensors could directly characterize the spatial and temporal dynamics of opioid release during cold stimulation. Such measurements, ideally combined with *Penk*-specific manipulation of lPBN^cold^ neurons, would further distinguish lPBN^cold^ neuron–derived enkephalin signaling from signals generated by other endogenous opioid peptides.

The aforementioned experimental results suggest that cold-induced analgesia is primarily mediated by the central nervous system, particularly supraspinal structures but not the peripheral level (fig. S2). However, others demonstrated that the formalin-induced spontaneous calcium response in DRG can be inhibited by topical menthol application and physical cooling inhibits spontaneous calcium responses in injured trigeminal ganglion neurons (*74*, *75*). This might contribute to the decreased activity of lPBN^HP^ neurons upon cold exposure (Fig. 2G). Moreover, recent studies have shown that activation of cold-responsive Kcnip2^+^ neurons in the spinal cold could induced analgesia (*76*), suggesting possible intraspinal local inhibitory processing in TRPM8-dependent analgesia. Previous research has reported that intrathecal injection of a group II/III mGluR inhibitor, but not naloxone, abolished TRPM8-dependent analgesia (*28*). This body of evidence suggests that TRPM8-dependent analgesia may be mediated at multiple levels, from the peripheral nervous system, spinal cord, and brainstem. In conclusion, our study elucidates the role of the lPBN-to-vlPAG neural circuit and enkephalin-dependent opioid signaling in TRPM8-dependent analgesia, highlighting a potential central target for novel pain therapeutics.

## MATERIALS AND METHODS

### Animals

All behavioral studies and primary culture except the TRAP studies were performed with 5- to 7-week-old male C57BL/6J wild-type mice (Doo Yeol Biotech, South Korea). For TRAP experiments, 6- to 8-week-old male TRAP2 mice (Fos^2A-iCreER^, JAX strain no. 030323) were used and crossed with reporter mice (Ai6, JAX strain no. 007906, or Ai9, JAX strain no.007909). They were backcrossed with C57BL/6J mice for more than 10 generations and were thought of as congenic to C57BL/6J. All animals were maintained in constant temperature (22 ± 1°C), humidity (55%), and 12/12-hour light/dark cycle environment with standard lab chow and water available ad libitum. Handling and care of the animals were in accordance with the Guideline for Animal Experiments, 2000, edited by the Korean Academy of Medical Sciences, which is consistent with the National Institutes of Health (NIH) Guidelines for the Care and Use of Laboratory Animals, revised 1996. All experimental procedures were approved by the Institutional Animal Care and Use Committee (IACUC) at Seoul National University (protocol code: SNU-241105-5).

### Primary culture of DRG neurons

Primary culture of DRG neurons was performed as previously reported (*77*). Briefly, after the mouse was euthanatized with isoflurane, DRGs were acutely dissected on ice-cold Hanks’ balanced salt solution (HBSS) with 20 mM Hepes (Gibco, no. 14025092). Then, ganglia were digested for 1 hour in collagenase A (1 mg/ml, Roche) and dispase II (2.4 U/ml, Roche) mixture in HBSS-Hepes–buffered solution in a 37°C incubator with 5% CO_2_. Then, 0.25% trypsin (Gibco, no. 15090046) in HBSS was applied for 7 min. DRGs were mechanically dissociated with trituration by a fire-polished Pasteur pipette in Dulbecco’s modified Eagle’s medium containing deoxyribonuclease I. The cell suspension was then carefully layered over 15% bovine serum albumin (BSA) in Ham’s F-12 (Welgene, no. LM010-52) to isolate neurons. Cells were resuspended with neurobasal medium (Gibco, no. 21103049) containing B-27 supplement (Gibco, no. 17504044), l-glutamine (Sigma-Aldrich, no. G7513), and penicillin-streptomycin (Gibco, no. 10348016). Three thousand DRG neurons were plated on a 10-mm-diameter coverslip coated with poly-d-lysine (100 μg/ml) for calcium imaging experiments.

### Ratiometric calcium imaging in primary sensory neurons

Cultured DRG neurons or transfected human embryonic kidney 293 cells were used for calcium imaging after incubation overnight. Cells were loaded with 2 μM Fura-2 AM in Dulbecco’s modified Eagle’s medium for 40 min in a 37°C incubator with 5% CO_2_. Images were acquired through a charge-coupled device camera (Andor Technology, DL-604M-VP) coupled to an inverted microscope (Nikon, Eclipse TE-2000), while an ultraviolet light source (Sutter Instruments, Lambda DG-4 plus) was used to excite Fura-2 by alternating 340- and 380-nm wavelength lights. Mean fluorescence intensity ratios (*F*_340_/*F*_380_) were measured with MetaFluor software. A bath solution (2 mM Ca^2+^-HBSS containing 140 mM NaCl, 5 mM KCl, 1 mM MgCl_2_, 2 mM CaCl_2_, 10 mM Hepes, and 10 mM glucose) was adjusted to pH 7.4 with 5 M NaOH and to 300 ± 5 mosm.

### Behavioral test

#### von Frey test

Before the behavior test, mice were habituated for at least three consecutive days to the test chamber and randomized for treatment. All sensory testing was performed between the hours of 09:00 and 18:00 in an isolated room maintained at 22 ± 2°C and 50 ± 10% humidity. For mechanical threshold (von Frey filament) testing, mice were brought from the home cage and placed in transparent plastic boxes on a metal mesh floor. The mice were then habituated for at least 30 min before testing. To assess mechanical sensitivity, the withdrawal threshold of the affected hindpaw was measured using a series of von Frey filaments (0.20, 0.40, 0.70, 1.6, 3.9, 5.9, 9.8, and 13.7 mN; Stoelting, Wood Dale, IL; equivalent in grams to 0.02, 0.04, 0.07, 0.16, 0.40, 0.60, 1.0, and 1.4). The 50% withdrawal threshold was determined using the “up-down” method. A brisk hindpaw lift, licking, or flinch in response to von Frey filament stimulation was regarded as a withdrawal response. The 0.4-g filament was the first stimulus to be used, and when a withdrawal response was obtained, the filament with the next lower number was used. This process was repeated until no response was obtained, at which time the filament with the next higher number was administered. All behavioral testing was performed by a blinded investigator who was blind to the treatment of the mice. For the CFA-induced inflammation model, 20 μl of CFA (Sigma-Aldrich, no. F5881) was injected in the ipsilateral hindpaw 1 day before the behavioral test.

#### Drug treatment

CNO was stored as a stock solution (10 mg/ml in saline, Tocris, no. 4936) and stored at −20°C. Before CNO-induced chemogenetic neuronal activation and/or inactivation experiments, the stock solution was thawed and diluted to the desired concentration with sterile saline. For intraperitoneal injection, the chemogenetic activation dose was set as 1 mg/kg, unless otherwise noted, and a CNO solution was given with a volume of 10 μl/g body weight. The chemogenetic inactivation dose was set as 5 mg/kg, unless otherwise noted, and a CNO solution was given with a volume of 10 μl/g body weight. For cannula-based local injection of CNO, 1 mM CNO dissolved in saline was used. Under light isoflurane anesthesia (1.5%, within 5 min), 500 nl of CNO solution was filled into a microsyringe (Trajan Scientific, no. 002050) and injected through a guide cannula with a syringe pump (KdScientific, no. KDS100) with a rate of 500 nl/min. The syringe needle tip was retained in place for at least 1 min before removal to prevent leakage. CNO injection was performed at least 15 min before the behavioral test, considering the pharmacokinetics of CNO in the central nervous system. CNO treatment was randomly counterbalanced with saline to prevent any potential bias on the basis of treatment order. For naloxone-mediated local block experiments, 5 μg of naloxone (Tocris, no. 0599) was co-injected with CNO at a volume of 500 nl.

#### Topical icilin treatment and cooling-induced analgesia

Icilin (Tocris, no. 1531) was stored as a stock solution of 10 mM in DMSO (dimethyl sulfoxide) and stored at −20°C. On the day of treatment, icilin was diluted to vehicle (2% Tween 80 in sterile saline) at a concentration of 200 μM. For topical icilin treatment, mice were lightly restrained, and an icilin solution (or vehicle for control) was applied twice to the bilateral hindpaw with cotton tips, each in 1 min. For icilin treatment in the tail, tails of mice were immersed in the icilin solution twice, each in 1 min. For cooling-induced analgesia, mice were placed on a wet metal plate set at 17°C (for cold treatment group) or 25°C [for RT (room temperature) treatment group] for 5 min before the behavioral test. After cold exposure, moist from the hindpaw was gently removed, and the behavior test was performed within 10 min after cold exposure.

#### Hot and cold plate assay

To measure the noxious thermal pain threshold, mice were placed onto a metallic hot plate or custom-made cold plate within an acrylic container. The temperature of the hot plate was set at 55°C, while that of the cold plate was set at −5°C. For the hot plate, the latency to nocifensive behavior (hindpaw licking, flinching, and jumping) was measured three times and averaged. A cutoff time of 30 s was used to prevent permanent tissue damage. For the cold plate, the latency to nocifensive behavior (hindpaw licking, flinching, and jumping) was measured two times and averaged. A cutoff time of 60 s was used to prevent permanent tissue damage. For nocifensive score measurement for the cold plate, mice were placed onto the cold plate, and nocifensive behaviors were counted for 30 s. Every bout of hindpaw licking, flinching, and jumping behavior was scored 2 points, and every bout of rearing, hindpaw lifting, and trial for chamber avoidance (digging-like behavior) was scored 1 point.

#### Thermal gradient test

Cooling and heating devices were used to maintain a stable temperature so that the surface temperature range of the apparatus was from 5° to 55°C in a linear manner. The floor surface temperature was monitored using an attached thermometer. Mice were acclimated for 30 min in the thermal gradient apparatus with its floor at room temperature before the day of the thermal gradient test. A camera is located upon the apparatus to record the position of mice in real time (60 frames per second). Mice were placed individually in the device in an innocuous middle zone, and the spontaneous movement of mice was videotaped for 30 min. The recorded video was analyzed with ezTrack to measure the travel distance or spent time in each region (or temperature zone) (*78*). The noxious cold zone (nCold) was defined as the zone with a temperature of 17°C or lower, the cool zone was defined as the zone with a temperature between 17° and 25°C, and the noxious hot zone (nHot) was defined as the zone with a temperature of 42°C or higher. The “preference temperature” was calculated with the average value using the zone temperature and “spent time.”

### Adhesive tape removal test

Adhesive tape removal test was performed as previously described (*79*). Briefly, after habituation of the animal to the test chamber, a patch of adhesive tape (3 mm by 4 mm) was attached at a central hairless part of the left forepaw. After tape placement, the animal was placed in the test chamber, and the latency to tape contact and tape removal was measured with a timer. The contact time is defined as the point that the mouse reacts to the presence of the adhesive tape strips with its paw or mouth. Experiments were repeated two times with at least 10 min of interval, and then the latency obtained from two tests was averaged. If the animal urinates and walks on its micturition before attempting to remove the adhesive tape, the session was immediately aborted, and a subsequent additional trial was performed after 10 min.

### Core body temperature measurement

Under light isoflurane anesthesia (1.5%, within 5 min), a rectal probe covered with Vaseline was slowly and carefully inserted to the rectum of the animal to prevent damage. After 30 s of thermal balancing, the core body temperature was measured for 1 min and averaged. To measure the core body temperature of animals with CNO-induced activation of lPBN^Cold^ neurons, CNO was administered 15 min before measurement. Control animals were given the same dose of saline. CNO and saline treatment was counterbalanced.

### 4-OHT treatment

Homozygote TRAP2 mice (Fos2A-iCreER, the Jackson Laboratory, no. 030323) or TRAP2 mice crossed with Ai6 mice (RCL-ZsGreen, the Jackson Laboratory, no. 007906) were used for experiments. Three to 4 weeks after stereotaxic adeno-associated virus (AAV) injection (in the case of AAV injection) or at the age of 6 to 8 weeks (in the case of TRAP2; Ai6 mice), mice were handled and treated with saline for acclimation for five consecutive days. On the day of treatment, mice were placed on a 17°C metal plate powered with Peltier devices for 1 hour for cold TRAP or left at room temperature for control TRAP. Immediately after cold exposure, 4-hydroxytamoxifen (4-OHT; 20 mg/kg, dissolved in 2% Tween 80 and 5% DMSO in saline, Sigma-Aldrich, no. H6278) was intraperitoneally injected.

### Stereotaxic surgery and cannula implant

During isoflurane anesthesia (1.5 to 2%), the disinfected scale of mice was cut open after shaving. A drop of hydrogen peroxide was applied to remove the periosteum, and then a 0.5-mm burr hole was made with a hand drill. A glass micropipette pulled from a glass capillary with a digital puller was filled with mineral oil and AAV solution (>10^13^ vg/ml, Addgene; detailed information for the AAV construct is listed in table S3) at the end. After slow insertion of a micropipette, the AAV solution was injected at lPBN [anteroposterior (AP): −5.00; mediolateral (ML): ±1.27; dorsoventral (DV): −3.27; mm from bregma], vlPAG (AP: −4.60; ML: ±0.50; DV: −2.60; mm from bregma), or RVM (AP: −5.80; ML: 0.00; DV: −6.00; mm from bregma) at a rate of 50 nl/min with WPI microinjection (WPI, Nanoliter-2020). The micropipette was withdrawn after 5 to 10 min. The scalp was sutured, and then mice were allowed for recovery and gene expression for >3 weeks. For GRIN lens insertion surgery for in vivo calcium imaging, a 400-μm-diameter optic cannula (RWD, no. R-FOC-L400C-50NA) was slowly inserted 250 μm above the lPBN. Thirty minutes after insertion, the cannula was removed, and the GRIN lens was inserted slowly at a speed of 200 μm/min using a custom holder connected to vacuum, which was attached to a stereotaxic arm to hold the GRIN lens (inscopix, no. 1050-004597) in place. After complete insertion, the GRIN lens was attached to the skull surface with dental cement and bone wax. To improve the adhesion of dental cement to the skull, the skull surface was scored with a scalpel and chemically etched with phosphoric acid gel for 30 s and completely dried before cement application. A guide cannula (26G, bilateral 6-mm cannula, Protech, no. C235-1.0) to vlPAG (AP: −4.60; ML: ±0.50; DV: −2.60; mm from bregma) was implanted with the same protocol.

### In vivo calcium imaging

After GRIN lens implantation, a custom holder was used to mount the UCLA miniscope (version 4.4) to the stereotaxic holder. The miniscope was placed with the right imaging window, and the baseplate was attached with dental cement and cured for 30 min. After complete curing, the miniscope was removed, and a protective cap was inserted. Mice were injected with dexamethasone (1 mg/kg) to prevent inflammation and swelling for five consecutive days after lens implantation and baseplating surgery. After 5 days of dexamethasone treatment, the GRIN lens surface was cleaned with lens paper socked with acetone and ethanol, and then the imaging window was checked again. If a clear image of cells could not be observed because of sustained inflammation in imaging windows or distortion of dental cement during curing was observed, mice were excluded from further functional imaging. For calcium imaging, mice were rightly anesthetized with isoflurane (1.5%), and the miniscope was mounted and focused. After full recovery from anesthesia, mice were lightly restrained with a head bar, and tails of mice were exposed to heat (55°C water), mechanical pinch, and cold (4°C water). The cellular response was recorded at a frame rate of 20 frames per second, and the raw video was analyzed with the Minian pipeline (*80*). The vignetting-corrected, denoised, background-removed, and motion-corrected video underwent the constrained nonnegative matrix factorization (CNMF) algorithm to estimate the temporal and spatial matrix. Last, cells were manually assessed to exclude any artifacts. Cellular calcium activity was clustered by the Euclidean distance and labeled according to the neural response pattern through custom R script.

### Immunohistochemistry

For tissue preparation, mice underwent trans-cardiac perfusion with ice-cold phosphate-buffered saline (PBS) and 4% paraformaldehyde in PBS. For cold-induced met-enkephalin immunoreactivity measure in vlPAG, mice were perfused and fixed with 4% paraformaldehyde in PBS within 5 min after cold stimulation. The harvested brain and spinal cord tissues were fixed overnight in 4% paraformaldehyde in PBS and cryopreserved with 30% sucrose in PBS for at least 48 hours. The brain and spinal cords were embedded in OCT compound, frozen, and cryosectioned by 30 μm per section with a cryotome. Sections were blocked and permeabilized with 4% normal horse serum in PBS containing 0.3% Triton-X for 1 hour at room temperature. Sections were washed with PBS, three times for 5 min each; then stained with primary antibodies overnight at room temperature; washed as mentioned above; and stained with secondary antibodies for 2 hours at room temperature. All sections were examined and counterstained with DAPI (1 μg/ml in PBS) for 10 min, washed three times with PBS, then mounted on a slide with mounting medium, covered with a coverslip, and sealed with nail polish. Slides were imaged with an Axioscan Slide scanner and analyzed with Zen blue edition, ImageJ, and QuPath. Detailed information for the antibody used in this study is shown in table S4. For IB4 staining, IB4-Alexa Fluor 488 Conjugate (1:500, Invitrogen, I21411) was coapplied with the primary antibody.

### Preparation of acute brain slices for single-cell suspension

Live, single-cell suspensions of brain slices were prepared as described, with a minor modification (*33*, *81*). Four or five male TRAP2; Ai6 mice underwent cold-TRAP procedures, and one control wild-type mouse was used for each experimental batch. All mice were anesthetized with sodium pentobarbital (50 mg/kg) in saline and perfused with an ice-cold artificial cerebrospinal fluid (aCSF; containing 92 mM NMDG, 2.5 mM KCl, 1.25 mM NaH_2_PO_4_, 30 mM NaHCO_3_, 20 mM Hepes, 25 mM glucose, 2 mM thiourea, 5 mM Na-ascorbate, 3 mM Na-pyruvate, 0.5 mM CaCl_2_·2H_2_O, and 10 mM MgSO_4_·7H_2_O; pH was titrated to 7.3 or 7.4 with HCl, and the solution was continuously bubbled with 95% O_2_ and 5% CO_2_ for at least 20 min before use). Brains were rapidly dissected, rested in ice-cold aCSF for 2 min, cut, and mounted to a vibratome with superbond, and coronal slices with a thickness of 350 μm were prepared using a vibratome from bregma −4.9 to −5.4 mm. Slices were recovered in aCSF containing 500 nM tetrodotoxin, 10 μM AP-V, and 10 μM 6,7-dinitroquinoxaline-2,3-dione (DNQX) for 30 min, and lPBN was removed with tissue punches, digested with pronase (1 mg/ml) for 50 min at 30°C with routine inverting, and mechanically dissociated with a pasture pipette (inner diameter, 250 μm) in aCSF containing 0.05% BSA. Cell suspensions were filtered twice through a cell strainer tube with a pore size of 35 μm in a 16°C centrifuge (660*g*, 3 min). Cell pellets were suspended with aCSF containing 0.05% BSA and 1 μg/ml DAPI for 15 min for DAPI staining and washed with aCSF containing 0.05% BSA. Remaining cell pellets were resuspended in aCSF containing 0.05% BSA for FACS.

### RNA sampling and sequencing analysis

The single-cell suspension was sorted with a FACSAria III cell sorter (BD biosciences) with a 70-nm nozzle. Forward scattering with area (FSC-A) and side scattering with area (SSC-A) parameters were used to isolate cells from debris, and singles were gated with forward scattering with area and width (FSC-A and FSC-W, respectively). After singlet gating, the detection window of DAPI and ZsGreen fluorescent signals was set with the single-cell suspension from wild-type mice, and fluorescent gates were compensated with single- and double-stained samples for each experiment. According to the gating strategy, ZsGreen^+^, DAPI^−^ cell populations (Zsg^+^ viable population) and ZsGreen^−^, DAPI^−^ cell populations (Zsg^−^ viable population) were sorted into ice-cold aCSF containing 0.05% BSA. After sorting, the collection tube was centrifuged at 660*g* for 5 min, and the supernatant was removed. Remaining cell pellets were dissolved with 2 ml of TRIzol solution and stored at −80°C before RNA isolation. Total RNA was isolated using TRIzol reagent (Invitrogen) or Maxwell RSC miRNA from kits (Promega Corporation). The RNA quality was assessed by the Agilent 4200 TapeStation System (Agilent Technologies), and RNA quantification was performed using Qubit (Thermo Fisher Scientific Inc.). Only RNA samples with RNA integrity (RIN) higher than 7.0 were included in further procedures. For control and test RNAs, the construction of the library was performed using the QuantSeq 3′ mRNA-Seq Library Prep Kit FWD (Lexogen) according to the manufacturer’s instructions. Briefly, each total RNA was prepared, an oligo-dT primer containing an Illumina-compatible sequence at its 5′ end was hybridized to the RNA, and reverse transcription was performed. After degradation of the RNA template, second strand synthesis was initiated by a random primer containing an Illumina-compatible linker sequence at its 5′ end. The double-stranded library was purified by using magnetic beads to remove all reaction components. The library was amplified to add the complete adapter sequences required for cluster generation. The finished library is purified from polymerase chain reaction components. High-throughput sequencing was performed as single-end 75–base pair sequencing using NextSeq 500/550 (Illumina Inc.). A quality control of raw sequencing data was performed using FastQC. Sequenced reads were trimmed for adapter sequences and low-quality filtered using bbduk. Then, the clean reads were mapped to the reference genome using STAR (*82*). The quantification of reads was processed using HTSeq-count (*83*). The read counts were processed on the basis of the TMM + CPM normalization method using the Python “conorm” package. Data mining and graphic visualization were performed using ExDEGA (Ebiogen Inc., Korea). Genes were considered significantly enriched if they exhibited a fold change >2 and have *P* < 0.05 in Zsg-positive cells compared to Zsg-negative cells, while those with a fold change of <0.5 were regarded as significantly depleted.

### Preparation of acute brain slices for ex vivo electrophysiology

For acute brain slice preparation for ex vivo patch-clamp recording, mice were briefly anesthetized with isoflurane and decapitated. Brains were rapidly dissected, rested in ice-cold aCSF for 2 min, cut, and mounted to a vibratome with superbond, and coronal slices with a thickness of 250 μm were prepared using a vibratome in ice-cold sucrose-based dissection buffer (containing 212.7 mM sucrose, 26 mM NaHCO_3_, 10 mM glucose, 5 mM KCl, 10 mM MgCl_2_, 0.5 mM CaCl_2_, and 1.23 mM NaH_2_PO_4_; pH was titrated to 7.25 with continuous bubbling with 95% O_2_ and 5% CO_2_ for at least 20 min, and osmolality was maintained at 330 mosm). Slices were recovered in 28°C aCSF (containing 124 mM NaCl, 26 mM NaHCO_3_, 10 mM glucose, 5 mM KCl, 1.5 mM MgCl_2_, 2.5 mM CaCl_2_, and 1.23 mM NaH_2_PO_4_; pH was titrated to 7.25 with continuous bubbling with 95% O_2_ and 5% CO_2_ for at least 20 min, and osmolality was maintained at 305 mosm) for 30 min.

### Whole-cell patch-clamp recording in brain slices

After recovery, cold-sensitive neurons in the lPBN (expressing fluorescence reporter protein) were recorded with a Multiclamp 700B amplifier (Axon Instruments) and Digidata-1550B (Axon Instruments); glass micropipette electrodes were pulled from borosilicate glass capillaries (BF150-86-10, Sutter Instrument) with a P-700 microprocessor-controlled puller (Sutter Instruments) containing a K-gluconate–based intracellular solution (for current-clamp recordings; containing 123 mM K-gluconate, 18 mM KCl, 10 mM NaCl, 3 mM MgCl_2_, 2 mM ATP-Na_2_, 0.3 mM GTP-Na, and 0.2 mM EGTA; adjusted to pH 7.3 with 1 M KOH) or cesium-based intracellular solution (for voltage-clamp recordings; containing 130 mM Cs-MeSO_4_, 0.5 mM EGTA, 5 mM TEA-Cl, 8 mM NaCl, 10 Hepes, 1 mM QX-314, 4 mM ATP-Mg, 0.4 mM GTP-Na, and 10 mM phosphocreatine-Na_2_; adjusted to pH 7.3 with 1 M CsOH) in temperature-controlled aCSF (25°C). The resistance of glass micropipettes was kept at 3 to 5 MΩ. For the resting membrane potential recording, current clamp recording was performed, with the holding current held at 0 pA, 3 min after membrane rupture for internal solution substitution. For the spontaneous action potential recording, recording was performed in current clamp mode, while the holding current was held at 0 pA. Cells showing action potential firing during 60-s baseline recording periods were considered as spontaneous action potential firing neurons. For the CNO perfusion experiment, after the whole-cell recording configuration was successfully formed, we recorded 1 min of baseline period (during normal extracellular solution perfusion, 1.5 ml/min of perfusion rate), followed by CNO (10 μM in aCSF) bath perfusion. After CNO perfusion, we recorded for additional 20 min as the posttreatment washout condition. For the optogenetic lPBN^cold^ neuron terminal stimulation experiment, vlPAG slices were collected, and the vlPAG^CTB+^ neuron was visualized through green fluorescence. To record the membrane potential in this neuron, recording was performed in current clamp mode, while the holding current was held at 0 pA. Optogenetic blue light stimulation was delivered through a fiber-optic cable coupled to an optopatcher (A-M Systems) during whole-cell recording. During optogenetic light stimulation (30 s each), the light intensity was set to 10 mW using a digitalized light meter and the pulse width was set to 10 ms. The light pulse was controlled with Pulse Pal (Open Ephys) and triggered through analog output TTL signal from Digidata-1550B in a time-locked manner. For naloxone pretreatment, the vlPAG slice was incubated under naloxone (10 μM)–containing aCSF for at least 5 min. After data acquisition, the stimulation-induced change of membrane potential (ΔmV) was measured as the difference of membrane potential between the baseline (30 s before optogenetic stimulation) and the stimulation window (60 s after stimulation onset). The action potential firing frequency and average membrane potential were analyzed with Clampfit (version 10.7.0.3, Molecular Devices) in each time window. A heatmap diagram showing the stimulation-induced change of membrane potential was generated through custom R code to display the moving-averaged (500-ms window) membrane potential increase. Only increases in membrane potential exceeding 5 standard deviations above the baseline are displayed. To record postsynaptic currents (IPSCs and EPSCs) in the vlPAG^CTB+^ neuron, neurons were voltage clamped at 0 and −60 mV, respectively, before optogenetic stimulation was delivered. The latency of IPSCs and EPSCs was measured and consequently fitted to a Gaussian model with a baseline offset to estimate the mean latency and synaptic jitter. The parameters (mean and jitter) were estimated using the nonlinear least-squares regression algorithm. During whole-cell patch-clamp recording, cells with an access resistance (*R*_a_) higher than 30 MΩ and/or a membrane resistance lower than 250 MΩ were excluded from the analysis.

### In situ hybridization

The RNAscope multiplex v2 kit (Advanced Cell Diagnostics) was used to perform fluorescence in situ hybridization according to the manufacturer’s protocols. For tissue preparation, mice underwent trans-cardiac perfusion with ice-cold PBS treated with diethyl pyrocarbonate (DEPC) under anesthesia with sodium pentobarbital (55 mg/kg). The harvested brain tissues were embedded in OCT compound, rapidly frozen and cryosectioned by 12 μm per section, and stored at −80°C before in situ hybridization. On the day of the fluorescence in situ hybridization experiment, slides were directly fixed in 4% paraformaldehyde for 1 hour followed by washing with DEPC-treated PBS and dehydration in 50, 75, and 100% ethanol at room temperature. Tissue samples were then processed following the RNAscope Multiplex fluorescent reagent kit v2 protocol (Advanced Cell Diagnostics, document number UM323100). Slides were imaged with the Axioscan Slide scanner and/or LSM 980 confocal microscope (40×) and then analyzed with QuPath version 0.3.2. Detailed information for the fluorescence in situ hybridization probe used in this study is shown in table S5.

### Statistical analysis

The comparison between two groups was analyzed by Student’s *t* test (paired or unpaired), and the comparison between multiple groups was analyzed by analysis of variance (ANOVA) and Bonferroni’s post hoc tests or Dunnett post tests using Prism software (version 8.4.2, GraphPad). Detailed statistical methods and descriptive statistics are provided in supplementary tables. Statistical significance was set at *P* < 0.05, and all data are presented as the means ± SEM.
