# Supplementary material for: Brainstem enkephalinergic neural circuit underlying cold-induced pain relief in mice
Source: Sci Adv. 2026 Nov 6;12(45):eaea3106. doi: 10.1126/sciadv.aea3106 (PMC13742151; doi:10.1126/sciadv.aea3106)
Supplement: Supplementary file 1 [file sciadv.aea3106_sm.pdf]

Science Advances
AAAS

Supplementary Materials for

Brainstem enkephalinergic neural circuit underlying cold-induced pain relief in mice

Hayun Kim *et al*.

Corresponding author: Seog Bae Oh, odolbae@snu.ac.kr

Sci. Adv. **12**, eaea3106 (2026)
DOI: 10.1126/sciadv.aea3106

This PDF file includes:

Figs. S1 to S10
Tables S1 to S5

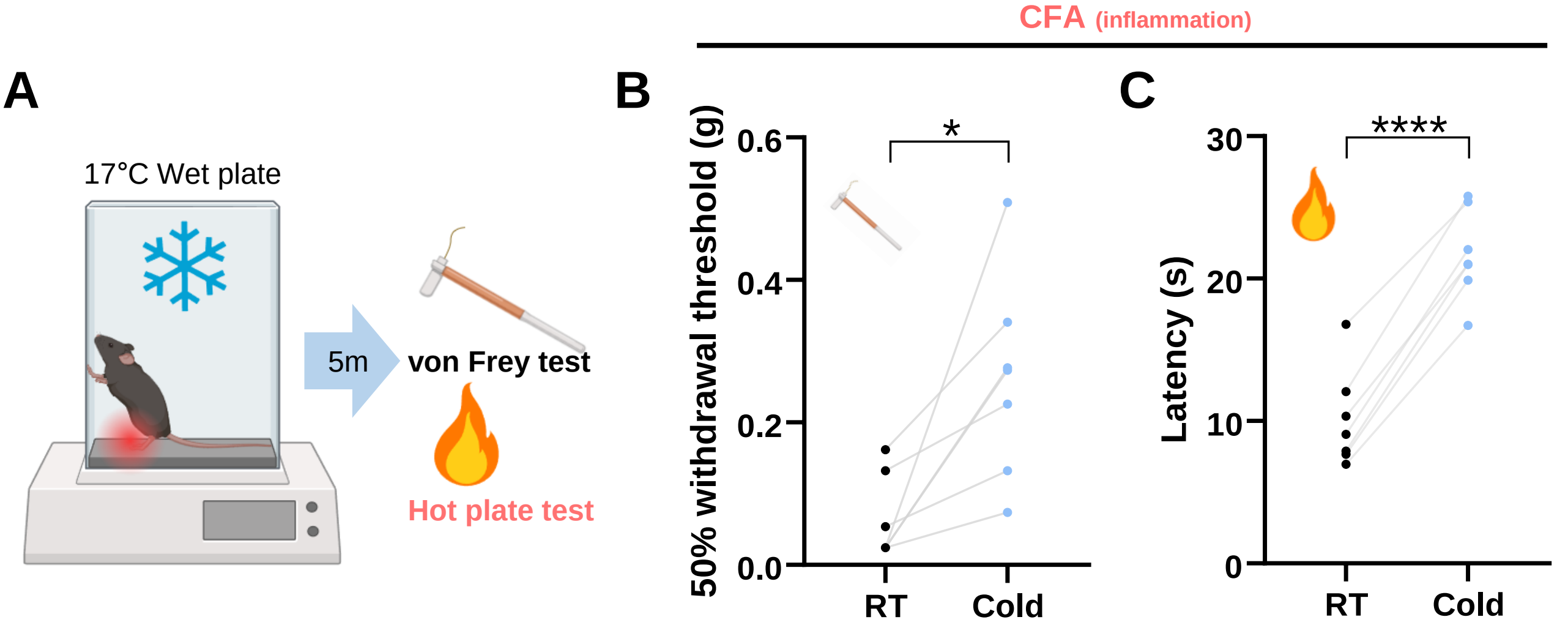

Figure S1: Effect of cold in CFA-induced mechanical allodynia and heat hyperalgesia
(**A**) Experimental scheme for the behavior test presented in Figure S1B-C (Created in BioRender. Oh. (2026) https://biorender.com/kfhjbry).
(**B**) Mechanical sensitivity measure with 50% withdrawal thresholds in CFA-induced inflammatory pain model mice in room temperature and cold treatment.
(**C**) Thermal sensitivity to noxious hot measured with response latency to hot plate in CFA-induced inflammatory pain model mice in room temperature and Cold treatment.
Data are presented as mean ± SEM. $*p < 0.05$, $**p < 0.01$, $***p < 0.001$ and $****p < 0.0001$. Number of mice and statistical tests are listed in **Table S2**.

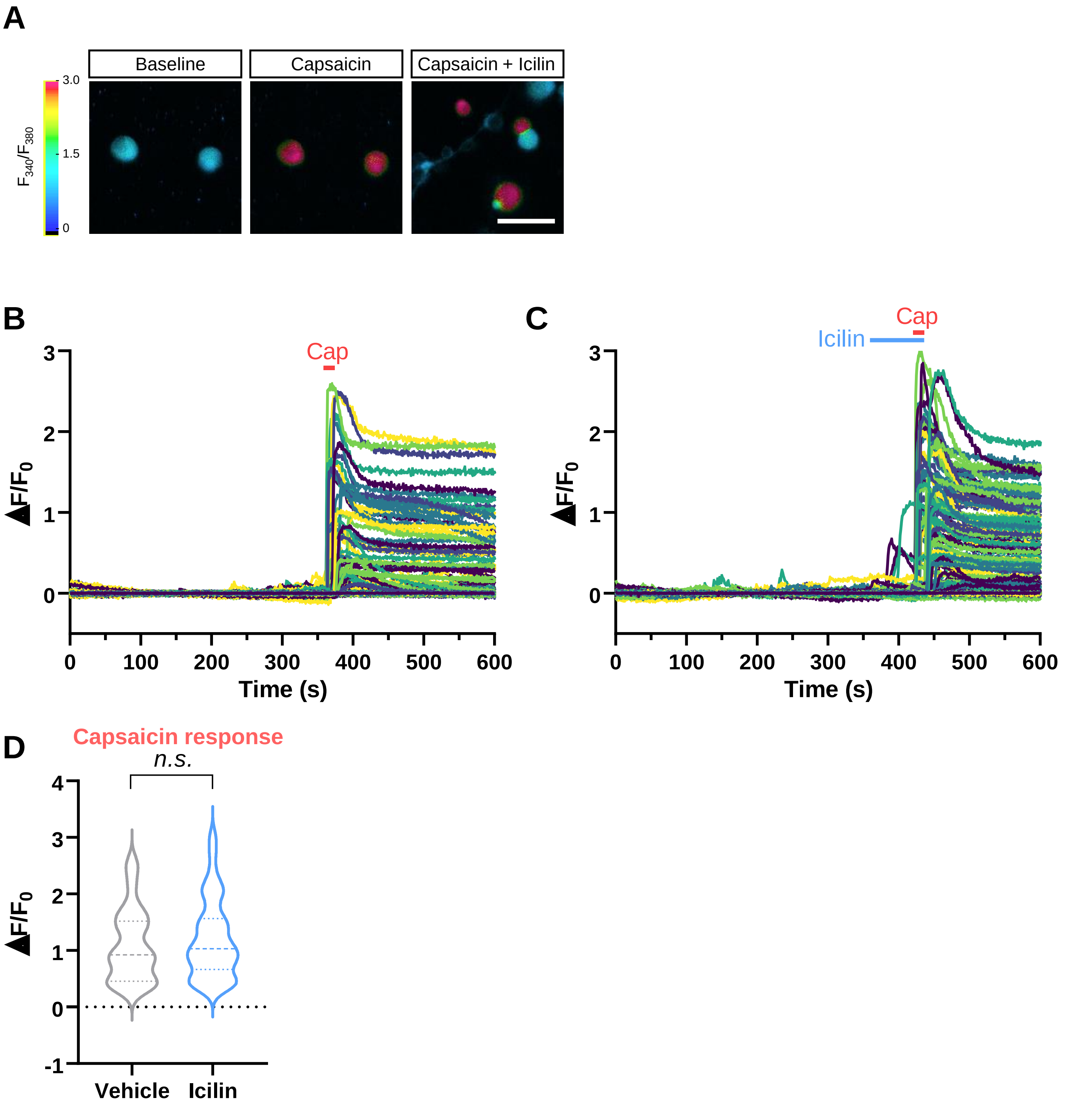

Figure S2: Effect of icilin on capsaicin-induced calcium response in peripheral sensory neurons

(**A**) Representative ratiometric image of Fura-2 calcium dye in primary cultured DRG neurons. Scale bar; 50μm.

(**B**) Representative calcium traces from naïve DRG neurons.

(**C**) Representative calcium traces from icilin-pretreated DRG neurons.

(**D**) Quantification of capsaicin-induced calcium response in DRG neurons.

Data are presented as mean ± SEM. $*p < 0.05$, $**p < 0.01$, $***p < 0.001$ and $****p < 0.0001$. Number of mice, cells and statistical tests are listed in **Table S2**.

A

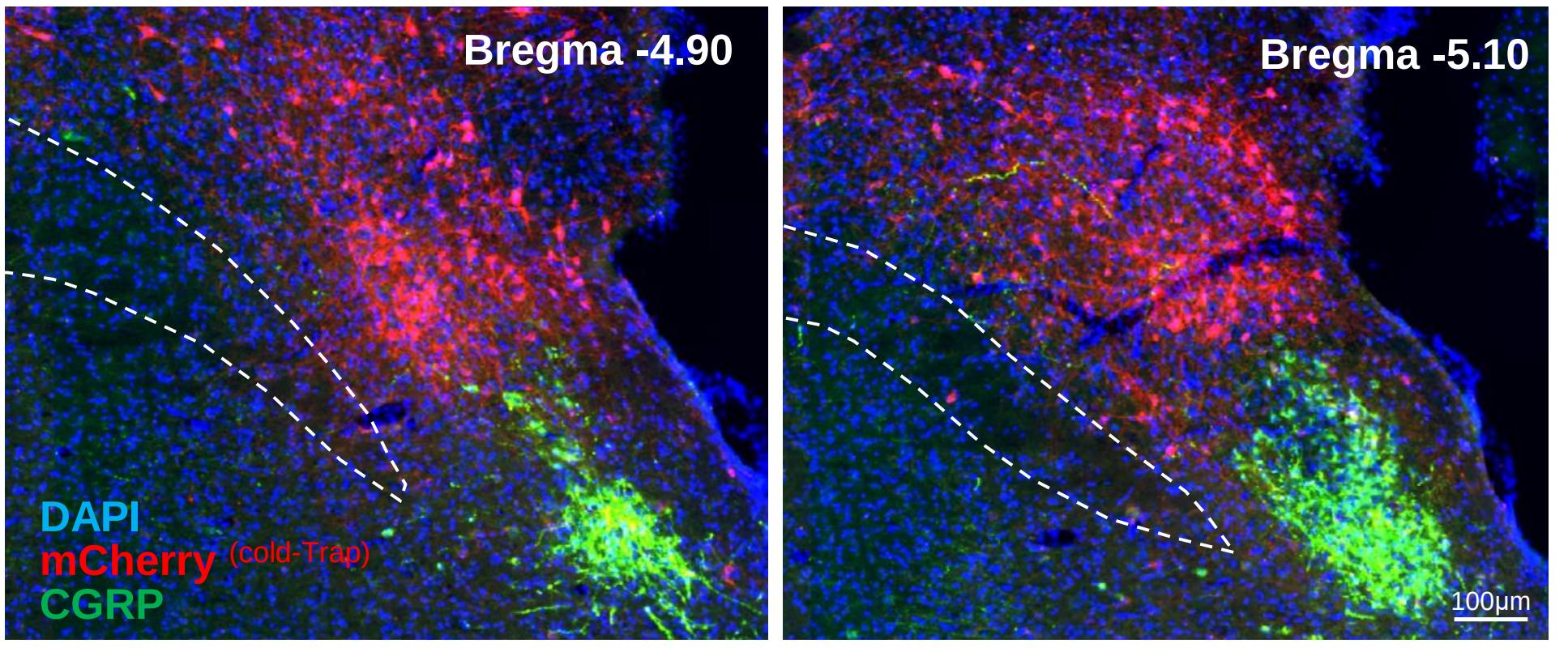

Figure S3: Lack of CGRP expression in lPBN^{cold} neurons
(**A**) Distribution of mCherry-positive (Cold-TRAP) neurons and anti-CGRP immunostaining in lateral parabrachial nucleus. Green; CGRP. Red; mCherry (cold-TRAP). Scale bar; 100µm.

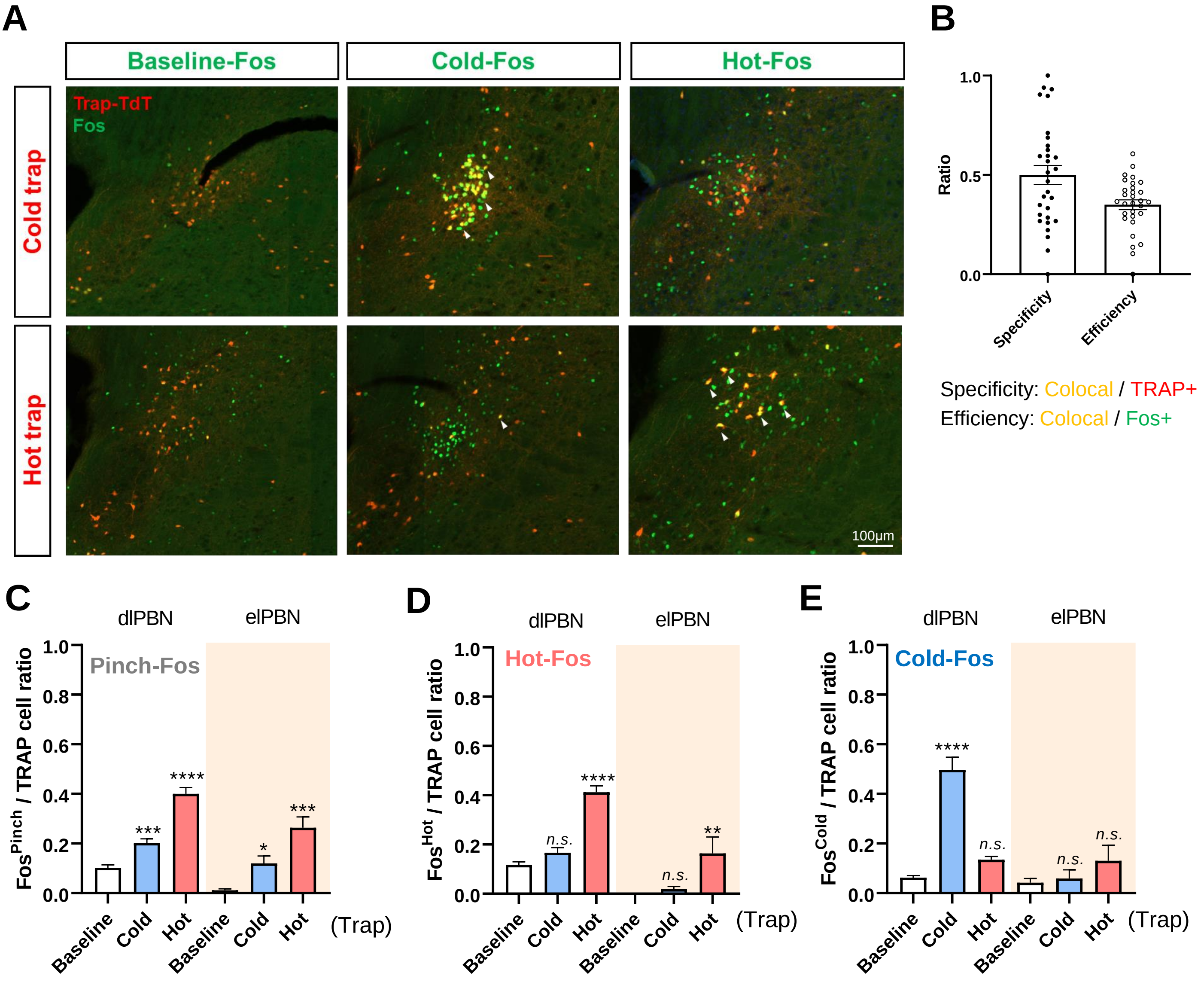

Figure S4: Double labeling experiments in lateral parabrachial nucleus for different stimuli

(**A**) Representative immunohistochemical images show neurons activated and labeled with TdTomato (TRAP signal) and c-Fos in the lateral parabrachial nucleus, with different stimuli. Scale bar; 100μm.

(**B**) Quantification of specificity and efficiency of TRAP methods.

(**C**) Ratio of pinch-Fos-positive cells in baseline-,cold-, and hot-TRAP lateral parabrachial neurons.

(**D**) Ratio of hot-Fos-positive cells in baseline-,cold-, and hot-TRAP lateral parabrachial neurons.

(**E**) Ratio of cold-Fos-positive cells in baseline-,cold-, and hot-TRAP lateral parabrachial neurons.

Data are presented as mean ± SEM. $*p < 0.05$, $**p < 0.01$, $***p < 0.001$ and $****p < 0.0001$. Number of mice and statistical tests are listed in **Table S2**.

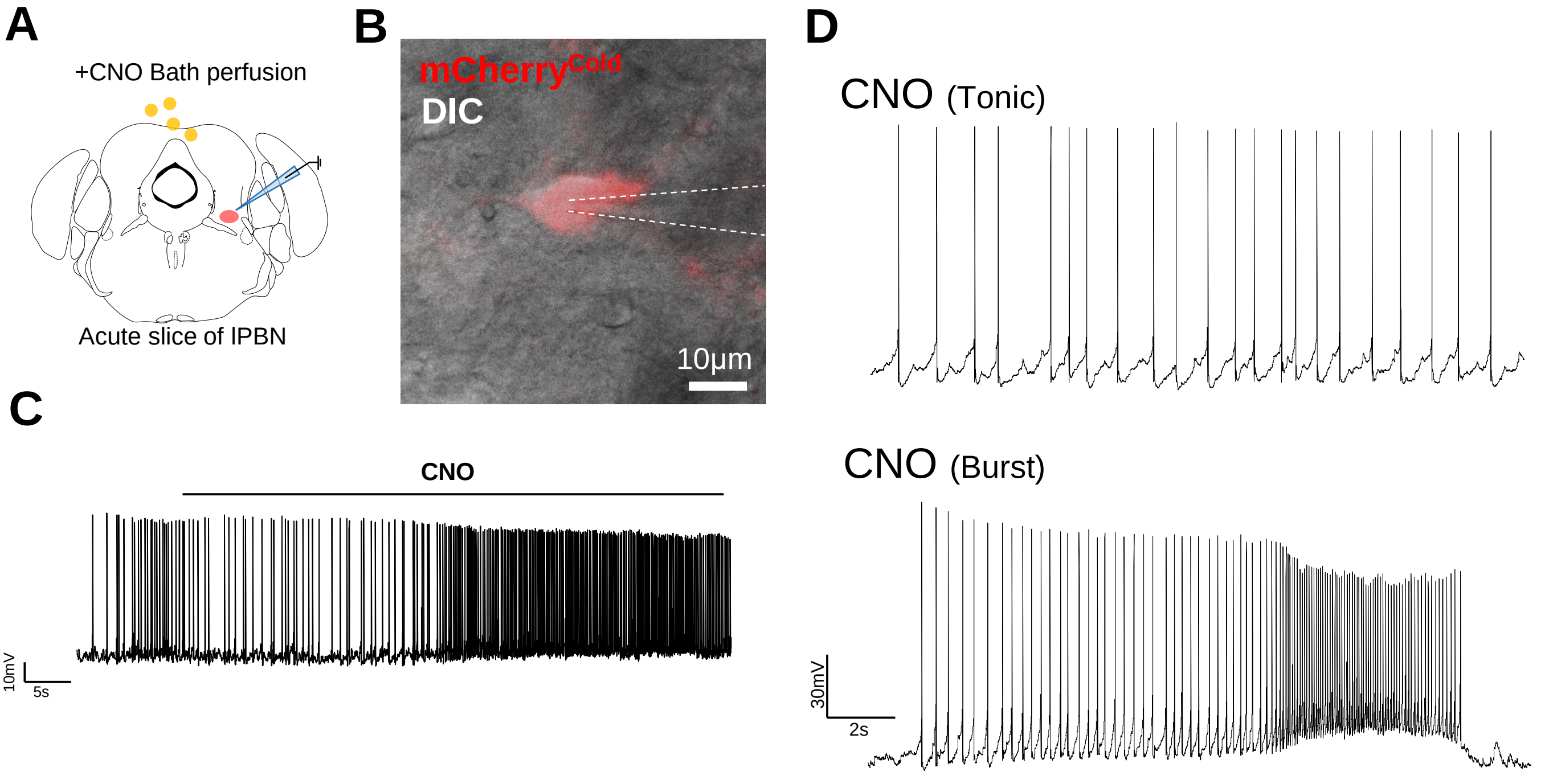

Figure S5: Electrophysiological validation of DREADD and characterization of lPBN^{cold} neurons

(**A**) Experimental scheme for the electrophysiological recording presented in Figure S5.

(**B**) Representative image of mCherry-positive lPBN^{cold} neurons. Red; mCherry. Images were reused from **Fig. 3A.**

(**C**) Representative membrane potential traces of lPBN^{cold} neurons during CNO treatment (10μM).

(**D**) Representative firing patterns of lPBN^{cold} neurons during CNO treatment (10μM).

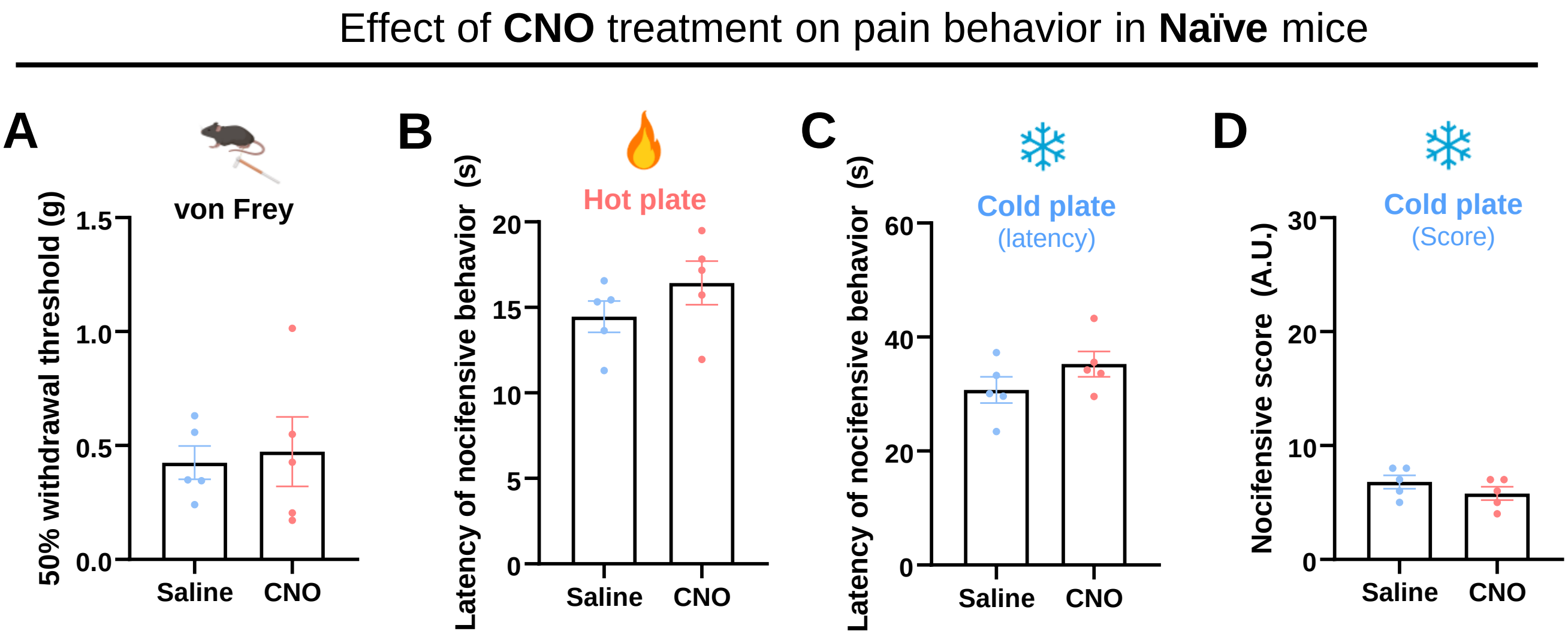

Figure S6: CNO treatment in naïve mice does not change baseline nocifensive behavior

(**A**) Mechanical sensitivity measured with 50% withdrawal thresholds in CNO-treated naïve mice (Created in BioRender. Oh. (2026) https://biorender.com/6r5yeg9).

(**B**) Thermal sensitivity to noxious hot measured with response latency to hot plate in CNO-treated naïve mice.

(**C**) Thermal sensitivity to noxious cold measured with response latency to cold plate in CNO-treated naïve mice.

(**D**) Thermal sensitivity to noxious cold measured with behavioral score to cold plate in CNO-treated naïve mice.

Data are presented as mean ± SEM. Number of mice and statistical tests are listed in **Table S2**.

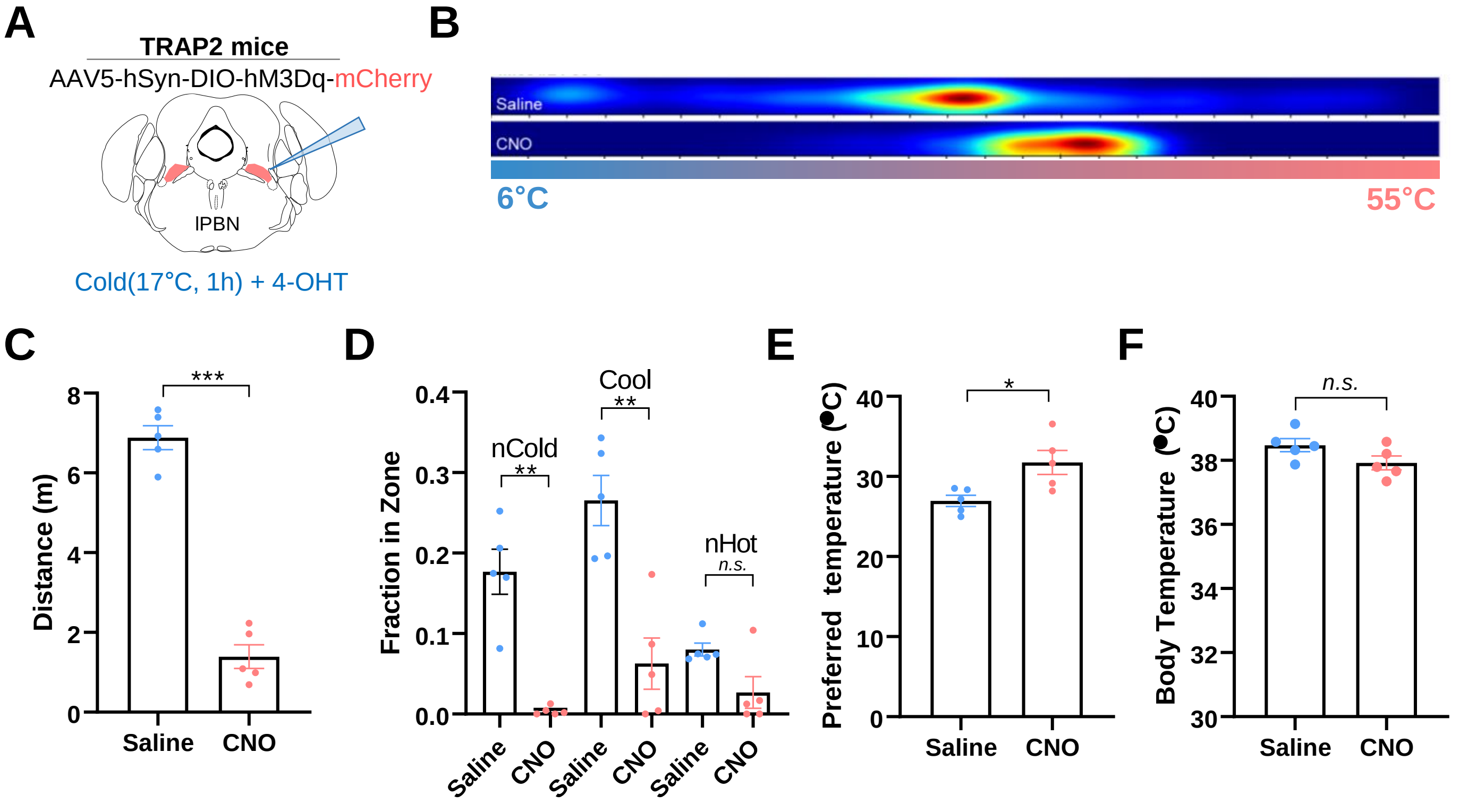

Figure S7: Activation of lPBN^{cold} neurons increases spontaneous cold avoidance

(**A**) Experimental scheme for the behavior test presented in Figure S7B-F.

(**B**) Heatmap of the thermal gradient test in saline-treated (top) and CNO-treated (bottom) mice.

(**C**) Total distance moved in thermal gradient test.

(**D**) Fraction of time spent in each Zone of thermal gradient plate (nCold; noxious cold, nHot; noxious hot).

(**E**) Preferred temperature of mice in thermal gradient test.

(**F**) Core body temperature of mice, measured with a rectal probe.

Data are presented as mean ± SEM. $*p < 0.05$, $**p < 0.01$, $***p < 0.001$ and $****p < 0.0001$. Number of mice and statistical tests are listed in **Table S2**.

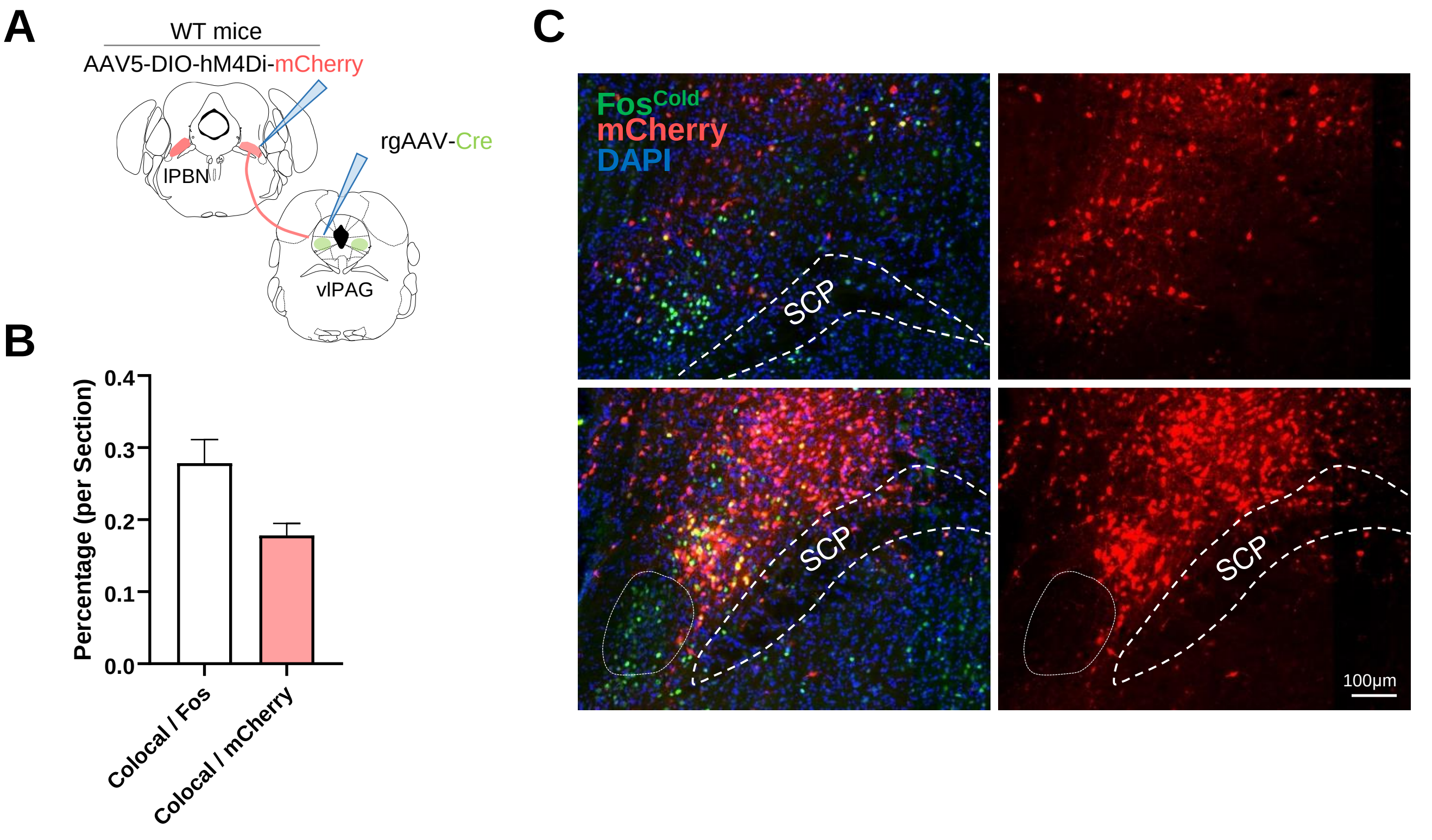

Figure S8: Activation of vlPAG-projecting lPBN neurons in response to cold

(**A**) Experimental scheme for the AAV-mediated tracing of vlPAG-projecting lPBN neurons.

(**B**) Quantification of cellular responsiveness.

(**C**) Representative immunohistochemical images show vlPAG-projecting lPBN neurons labeled with TdTomato and cold-induced c-Fos in the lateral parabrachial nucleus. Scale bar; 100μm.

Data are presented as mean ± SEM. Number of mice and statistical tests are listed in **Table S2**.

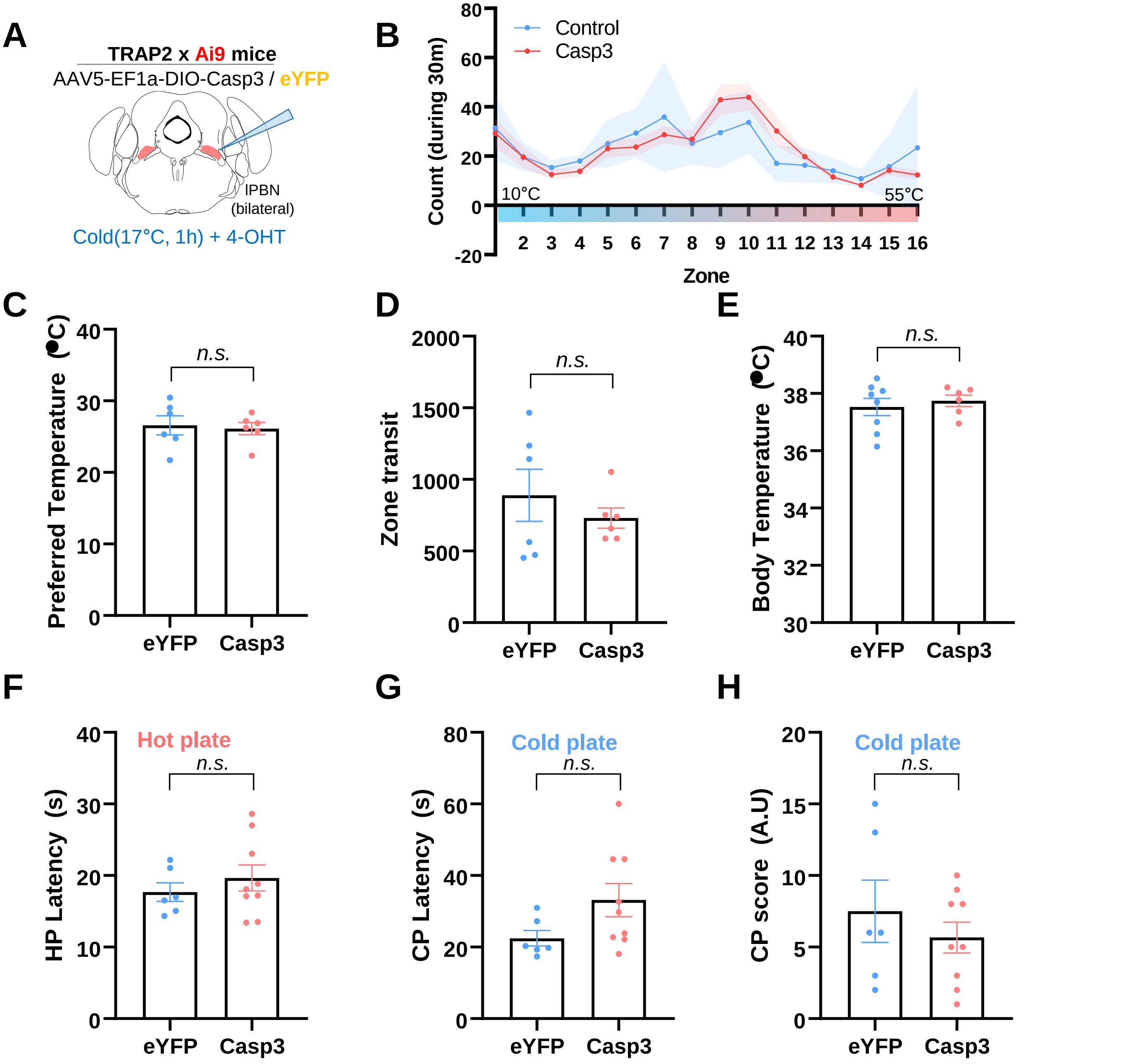

Figure S9: Ablation of lPBN^{cold} neurons does not affect thermal preference, basal body temperature and baseline nociception

(**A**) Experimental scheme for the AAV-mediated selective ablation of lPBN^{cold} neurons.

(**B**) Quantification of thermal gradient test in control mice (blue) and AAV-mediated ablation mice (red).

(**C**) Preferred temperature of mice in thermal gradient test.

(**D**) Total zone transit in thermal gradient test.

(**E**) Core body temperature of mice, measured with a rectal probe.

(**F**) Thermal sensitivity to noxious hot measured with response latency to hot plate in lPBN^{cold} neuron activation experiment.

(**G**) Thermal sensitivity to noxious cold measured with response latency to cold plate in lPBN^{cold} neuron activation experiment.

(**H**) Thermal sensitivity to noxious cold measured with nocifensive score to cold plate in lPBN^{cold} neuron activation experiment.

Data are presented as mean ± SEM. $*p < 0.05$, $**p < 0.01$, $***p < 0.001$ and $****p < 0.0001$. Number of mice and statistical tests are listed in **Table S2**.

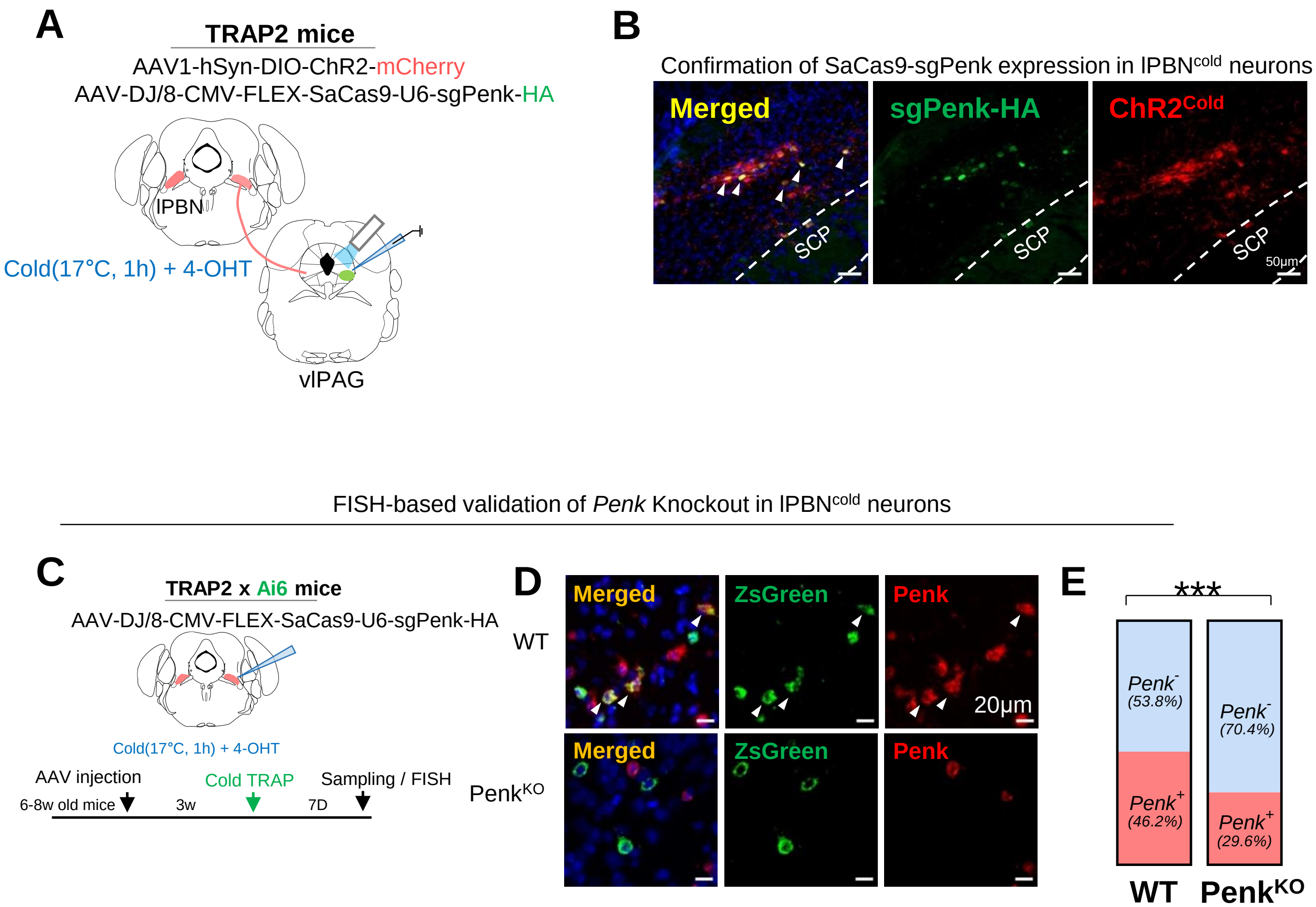

Figure S10: Confirmation of *Penk* knockout in lPBN^{cold} neurons

(**A**) Experimental scheme for AAV injection.

(**B**) Representative image of SaCas9-sgPenk expressing lPBN^{cold} neurons. SaCas9 was identified through HA epitope tag immunostaining. Red; ChR2-mCherry, Green; SaCas9-HA. Scale bar: 50µm.

(**C**) Experimental timeline for the cold-TRAP neuron validation.

(**D**) Representative image of FISH-based validation of *Penk* KO in lPBN brain sections. Red; *Penk*, Green; *ZsGreen* (cold-TRAP). Scale bar: 20µm.

(**E**) Quantification of Penk-positive neuronal population in lPBN.

Data are presented as mean ± SEM. $*p < 0.05$, $**p < 0.01$, $***p < 0.001$ and $****p < 0.0001$. Number of mice and statistical tests are listed in **Table S2**.

Supplementary Table 1. Number of mice/neurons and statistical tests in main figures.

Figure	Number of experimental objects	Statistical test	Descriptive statistics (Mean ± SEM)
1B	Vehicle and icilin, n = 15 mice each	Paired t-test, P = 0.0297	0.5880 ± 0.0574 for vehicle, 0.9460 ± 0.1310 for icilin
1C	Vehicle and icilin, n = 8 mice each	Paired t-test, P = 0.0027	0.1162 ± 0.031 for vehicle, 0.778 ± 0.173 for icilin
1D	Vehicle and icilin, n = 5 mice each	Paired t-test, P = 0.0113	0.0271 ± 0.0052 for vehicle, 0.287 ± 0.0573 for icilin
1E, bottom	Control TRAP, n = 3 mice; Cold TRAP, n = 7 mice	Paired t-test, P = 0.0033 for ACC, P = 0.485 for aIC, P < 0.0001 for pIC, P < 0.0001 for S1, P = 0.477 for PVT, P = 0.477 for DMH, P = 0.516 for VMH, P = 0.0166 for vlPAG	1.38 ± 0.179 for ACC, 1.03 ± 0.374 for aIC, 2.224 ± 0.654 for pIC, 2.096 ± 0.478 for S1, 0.909 ± 0.279 for PVT, 1.174 ± 0.338 for DMH, 0.823 ± 0.372 for VHM, 1.742 ± 0.421 for vlPAG (fold change)
1G, left	Control TRAP, n = 3 mice; Cold TRAP, n = 7 mice	Paired t-test, P < 0.0001 for -4.9mm, P = 0.805 for -5.1mm, P = 0.770 for -5.3mm	-4.9mm: 3.50 ± 0.69 for Control, 12.74 ± 1.42 for Cold / -5.10mm; 2.30 ± 0.60 for control, 2.10 ± 0.403 for Cold / -5.30mm; 0.53 ± 0.355 for control, 0.66 ± 0.245 for cold
1G, right	Control TRAP, n = 3 mice; Cold TRAP, n = 7 mice	Paired t-test, P = 0.838 for -4.9mm, P = 0.048 for -5.1mm, P = 0.282 for -5.3mm	-4.9mm: 15.67 ± 2.68 for Control, 14.91 ± 2.15 for Cold / -5.10mm; 17.40 ± 1.93 for control, 23.66 ± 1.62 for Cold / -5.30mm; 4.88 ± 1.37 for control, 6.83 ± 1.07 for cold
3C	n = 7 cells from 4 mice	One-Way ANOVA with Dunnett's multiple comparisons test, P = 0.0400 for Baseline vs CNO, P = 0.0665 for Bath vs washout.	0.531 ± 0.052 for Saline-treated, 1.494 ± 0.191 for CNO (0.1mg/kg) treated, 2.129 ± 0.221 for CNO (1mg/kg) treated
3E	n = 17 cells	One-Way ANOVA, P = 0.5220	-47.47 ± 1.361 for Total cell, -49.89 ± 2.67 for quiescent cell, -46.46 ± 1.557 for spontaneously active cell
3F	n = 10 mice for Saline-treated and CNO (1mg/kg) treated, n = 5 mice for CNO (0.1mg/kg) treated	One-Way ANOVA with Dunnett's multiple comparisons test, P < 0.0001 for Treatment; P = 0.164 for Saline vs CNO (0.1 mg/kg), P < 0.0001 for Saline vs CNO (1mg/kg)	0.535 ± 0.303 for baseline, 2.355 ± 0.850 for CNO, 0.996 ± 0.423 for washout
3G	n = 10 mice for Saline-treated and CNO (1mg/kg) treated, n = 5 mice for CNO (0.1mg/kg) treated	One-Way ANOVA with Dunnett's multiple comparisons test, P < 0.0001 for Treatment; P = 0.164 for Saline vs CNO (0.1 mg/kg), P < 0.0001 for Saline vs CNO (1mg/kg)	12.52 ± 1.03 for Saline-treated, 16.21 ± 1.29 for CNO (0.1mg/kg) treated, 19.60 ± 0.245 for CNO (1mg/kg) treated
3H	n = 15 mice for Saline-treated and CNO (1mg/kg) treated, n = 5 mice for CNO (0.1mg/kg) treated	One-Way ANOVA with Dunnett's multiple comparisons test; P = 0.532 for Saline vs CNO (0.1mg/kg), P = 0.048 for Saline vs CNO (1mg/kg)	27.65 ± 2.87 for Saline-treated, 22.56 ± 6.87 for CNO (0.1mg/kg) treated, 16.72 ± 3.41 for CNO (1mg/kg) treated
3I	n = 15 mice for Saline-treated and CNO (1mg/kg) treated, n = 5 mice for CNO (0.1mg/kg) treated	One-Way ANOVA with Dunnett's multiple comparisons test, P = 0.0046 for Treatment; P = 0.0404 for Saline vs CNO (0.1 mg/kg), P = 0.0014 for Saline vs CNO (1mg/kg)	6.87 ± 0.96 for Saline-treated, 17.80 ± 4.50 for CNO (0.1mg/kg) treated, 20.13 ± 3.31 for CNO (1mg/kg) treated
3J	n = 5 mice each	Paired t-test, P = 0.0159	0.0496 ± 0.028 for Saline-treated, 1.611 ± 0.371 for CNO treated
4D	n = 4 mice each	Paired t-test, P = 0.0393	0.480 ± 0.0091 for vehicle, 1.134 ± 0.106 for CNO
4E	n = 4 mice each	Paired t-test, P = 0.0184	18.47 ± 1.94 for vehicle, 26.15 ± 1.765 for CNO
4F	n = 4 mice each	Paired t-test, P = 0.0242	13.05 ± 2.77 for vehicle, 2.790 ± 0.835 for CNO
4G	n = 4 mice each	Paired t-test, P = 0.0017	14.50 ± 3.23 for vehicle, 37.50 ± 1.56 for CNO
4H, left	n = 4 mice each	Paired t-test, P = 0.486	11.10 ± 1.977 for vehicle, 14.31 ± 2.885 for CNO
4H, right	n = 4 mice each	Paired t-test, P = 0.0017	17.93 ± 2.13 for vehicle, 24.28 ± 9.09 for CNO
4I	n = 8 mice each	One-Way ANOVA with Dunnett's multiple comparisons test; P = 0.0486 for Saline vs CNO, P = 0.6858 for Saline vs CNO+Naloxone	0.468 ± 0.056 for Saline, 0.961 ± 0.145 for CNO, 0.628 ± 0.172 for CNO+Nal
4J	n = 5 mice each	One-Way ANOVA with Tukey's multiple comparisons test; P = 0.0095 for Baseline vs CFA-saline, P = 0.0.42 for CFA-saline vs CFA-CNO	0.569 ± 0.0087 for Baseline, 0.0497 ± 0.028 for CFA-Saline, 1.611 ± 0.370 for CFA-CNO
5C	n = 2 mice for Control, n = 3 mice for Casp3; 18 sections from Control; 30 sections from Casp3	Unpaired t-test, P < 0.0001	30.44 ± 3.29 for Control, 9.90 ± 1.87 for Casp3
5D	n = 6 mice for control, n = 9 mice for Casp3	Unpaired t-test, P = 0.917	0.630 ± 0.0237 for Control, 0.682 ± 0.078 for Casp3
5E	n = 5 mice each	Paired t-test, P = 0.5600	0.782 ± 0.056 for Baseline, 0.724 ± 0.074 for Vehicle
5F	n = 15 mice each for control, n = 8 mice each for Casp3	Paired t-test, P = 0.0297 for Control group, P = 0.7673 for Casp3 group	Control: 0.588 ± 0.057 for vehicle, 0.946 ± 0.131 for icilin; Casp3: 0.710 ± 0.051 for vehicle, 0.688 ± 0.067 for icilin
5G, control	n = 8 mice each	Two-Way ANOVA with Sidak's multiple comparison test, Baseline; P = 0.7304, CFA; P = 0.0022, CFA+icilin; P = 0.9768	Contralateral: 0.680 ± 0.134 for Baseline, 0.775 ± 0.133 for CFA, 0.844 ± 0.132 for CFA+icilin; Ipsilateral: 0.510 ± 0.076 for Basline, 0.116 ± 0.0288 for CFA, 0.777 ± 0.161 for CFA+icilin
5G, Casp3	n = 8 mice each	Two-Way ANOVA with Sidak's multiple comparison test, Baseline; P = 0.6995, CFA; P = 0.6485, CFA+icilin; P = 0.0100	Contralateral: 0.805 ± 0.105 for Baseline, 0.956 ± 0.226 for CFA, 0.876 ± 0.088 for CFA+icilin; Ipsilateral: 0.614 ± 0.077 for Basline, 0.307 ± 0.105 for CFA, 0.274 ± 0.107 for CFA+icilin
5H	n = 8 mice for control, n = 8 mice for Casp3	Unpaired t-test, P = 0.0341	0.6613 ± 0.146 for control, 0.020 ± 0.242 for Casp3
5J	n = 8 mice each	Paired t-test; P = 0.0411 for saline, P = 0.4041 for CNO	Saline: 0.412 ± 0.0638 for vehicle, 0.750 ± 0.122 for icilin; CNO: 0.425 ± 0.0383 for vehicle, 0.4923 ± 0.0778 for icilin
6B	n = 4 experimental batches for WT, n = 5 batches for TRAP-ZsGreen (each with 4-5 mice)	Unpaired t-test, P = 0.0082	0.2750 ± 0.103 for WT, 5.880 ± 1.354 for TRAP
6C	n = 5 batches	N.A.	79.34 ± 2.665 (%)
6I	n = 2 mice for Saline, n = 3 mice for CNO	Unpaired t-test, P = 0.0054	1.00 ± 0.034 for Control, 1.11 ± 0.0157 for cold
6J	n = 3 mice each	Unpaired t-test, P = 0.0414	1.00 ± 0.017 for Control, 1.054 ± 0.0175 for cold
6L	n = 8 mice for WT and lPBN^{cold} Penk KO, n = 5 mice for lPBN Penk KO	One-Way ANOVA with Tukey's multiple comparisons test; P = 0.0004 for WT vs lPBN Penk KO, P = 0.0007 for WT vs lPBN^{cold} Penk KO	0.6613 ± 0.146 for WT, -0.045 ± 0.0341 for lPBN Penk KO, 0.0741 ± 0.0324 for lPBN^{cold} Penk KO
7F	n = 11 cells for 5Hz, n = 11 cells for 40Hz, n = 6 cells for 40Hz+Nal; each from 3-4 mice	One-Way ANOVA with Tukey's multiple comparisons test; P = 0.0005 for 5Hz vs 40Hz, P = 0.1199 for 5Hz vs 40Hz+nal, P = 0.1738 for 40Hz vs 40Hz+Nal	-0.9648 ± 0.331 for 5Hz, 1.562 ± 0.3406 for 40Hz, 0.5044 ± 0.3825 for 40Hz+Nal
7G	n = 12 cells for 5Hz, n = 6 cells for 40Hz, n = 8 cells for 40Hz+Nal; each from 3-4 mice	One-Way ANOVA with Tukey's multiple comparisons test; P = 0.0071 for 5Hz vs 40Hz, P = 0.7481 for 5Hz vs 40Hz+nal, P = 0.0497 for 40Hz vs 40Hz+Nal	1.090 ± 0.04816 for 5Hz, 0.8209 ± 0.071 for 40Hz, 1.037 ± 0.0480 for 40Hz+Nal
7J	n = 10 cells each from 3-4 mice	Unpaired t-test, P = 0.9644	-0.0602 ± 0.302 for 5Hz, -0.0432 ± 0.2254 for 40Hz
7K	n = 10 cells each from 3-4 mice	Unpaired t-test, P = 0.0006	2.524 ± 0.4452 for WT, 0.0174 ± 0.421 for Penk^{KO}
8C	n = 4 mice each	Unpaired t-test, P < 0.0001	16.97 ± 1.296 for saline, 7.726 ± 0.807 for CNO
8D	n = 4 mice each	Unpaired t-test, P = 0.0005	4.436 ± 0.8275 for saline, 2.557 ± 0.4786 for CNO
8F	n = 3 mice for saline, n = 4 mice for CNO	Unpaired t-test, P = 0.0368	3.765 ± 0.298 for saline, 2.425 ± 0.222 for CNO
8G	n = 3 mice for saline, n = 4 mice for CNO	Unpaired t-test, P = 0.0002	5.739 ± 0.7885 for saline, 2.848 ± 0.3590 for CNO
8I	n = 4 mice each	Unpaired t-test, P < 0.0001	68.29 ± 2.566 for saline, 38.68 ± 1.368 for CNO
8J	n = 4 mice each	Unpaired t-test, P = 0.1069	5.597 ± 0.494 for saline, 4.50 ± 0.456 for CNO
9C	n = 5 mice each	Paired t-test, P = 0.0348	0.474 ± 0.063 for saline, 1.741 ± 0.387 for CNO
9D	n = 5 mice each	Paired t-test, P < 0.0001	16.66 ± 0.837 for saline, 29.73 ± 0.271 for CNO
9E	n = 5 mice each	Paired t-test, P = 0.2545	6.20 ± 2.15 for saline, 3.80 ± 1.16 for CNO
9H	n = 2 mice for Saline, n = 3 mice for CNO	Unpaired t-test, P < 0.0001	13.64 ± 0.569 for Saline, 6.05 ± 0.207 for CNO
9I	n = 2 mice for Saline, n = 3 mice for CNO	Unpaired t-test, P < 0.0001	14.76 ± 1.889 for Saline, 3.570 ± 0.3976 for CNO
9J	n = 2 mice for Saline, n = 3 mice for CNO	Unpaired t-test, P < 0.0001	35.47 ± 1.088 for Saline, 22.82 ± 0.5258 for CNO

Supplementary Table 2. Number of mice/neurons and statistical tests in Supplementary Figures.

Supple. Fig	Number of experimental objects	Statistical test	Descriptive statistics (Mean ± SEM)
1B	n = 7 mice each	Paired t-test, P = 0.0130	0.063 ± 0.0221 for RT, 0.261 ± 0.0537 for Cold
1C	n = 7 mice each	Paired t-test, P < 0.0001	10.11 ± 1.295 for RT, 21.68 ± 1.190 for Cold
2D	46 DRG neurons for Capsaicin only, 89 DRG neurons for Capsaicin + Icilin	Unpaired t-test, P = 0.4063	1.091 ± 0.0943 for Capsaicin only, 1.194 ± 0.0734 for Capsaicin + Icilin
4B	30 sections each, from 5 mice	N.A.	specificity of 0.500 ± 0.048, efficiency of 0.350 ± 0.135
4C	n = 5 mice each; 39 sections from baseline TRAP; 38 sections from Cold TRAP; 36 sections from Hot trap	One-Way ANOVA with Dunnett's multiple comparisons test, P < 0.0001 for Baseline vs Cold, P = 0.0004 for Baseline vs Hot (dlPBN); P < 0.0001 for Baseline vs Cold, P = 0.0180 for Baseline vs Hot (elPBN)	0.102 ± 0.012 for Baseline, 0.400 ± 0.0253 for Cold, 0.202 ± 0.017 for Hot (dlPBN); 0.011 ± 0.0061 for Baseline, 0.264 ± 0.043 for Cold, 0.120 ± 0.030 for Hot (elPBN)
4D	n = 4 mice each; 25 sections from baseline TRAP; 28 sections from Cold TRAP; 23 sections from Hot trap	One-Way ANOVA with Dunnett's multiple comparisons test, P = 0.1542 for Baseline vs Cold, P < 0.0001 for Baseline vs Hot (dlPBN); P = 0.8834 for Baseline vs Cold, P = 0.0022 for Baseline vs Hot (elPBN)	0.1172 ± 0.012 for Baseline, 0.1659 ± 0.021 for Cold, 0.412 ± 0.026 for Hot (dlPBN); 0 for Baseline, 0.0183 ± 0.011 for Cold, 0.1633 ± 0.067 for Hot (elPBN)
4E	n = 4 mice each; 28 sections from baseline TRAP; 30 sections from Cold TRAP; 22 sections from Hot trap	One-Way ANOVA with Dunnett's multiple comparisons test, P < 0.0001 for Baseline vs Cold, P = 0.2217 for Baseline vs Hot (dlPBN); P = 0.9244 for Baseline vs Cold, P = 0.2245 for Baseline vs Hot (elPBN)	0.042 ± 0.017 for Baseline, 0.058 ± 0.035 for Cold, 0.130 ± 0.063 for Hot (dlPBN); 0.062 ± 0.0084 for Baseline, 0.497 ± 0.051 for Cold, 0.135 ± 0.013 for Hot (elPBN)
6A	n = 5 mice each	Paired t-test, P = 0.7709	0.4229 ± 0.073 for Saline, 0.4731 ± 0.152 for CNO
6B	n = 5 mice each	Paired t-test, P = 0.2426	14.46 ± 0.0914 for Saline, 16.43 ± 1.272 for CNO
6C	n = 5 mice each	Paired t-test, P = 0.1949	30.73 ± 2.288 for Saline, 35.25 ± 2.236 for CNO
6D	n = 5 mice each	Paired t-test, P = 0.2598	6.80 ± 0.583 for Saline, 5.80 ± 0.583 for CNO
7C	n = 5 mice each	Paired t-test, P = 0.0004	6.880 ± 0.300 for Saline, 1.393 ± 0.298 for CNO
7D	n = 5 mice each	Paired t-test, P = 0.0028 (noxious cold Zone); P = 0.0099 (innocuous cold Zone); P = 0.0803 (noxious hot Zone)	Saline; 0.177 ± 0.028 for Noxious cold, 0.2654 ± 0.0312 for innocuous cold, 0.0802 ± 0.0081 for noxious hot; CNO; 0.0038 ± 0.0024 for Noxious cold, 0.0627 ± 0.032 for innocuous cold, 0.0267 ± 0.0197 for noxious hot
7E	n = 5 mice each	Paired t-test, P = 0.0188	26.96 ± 0.695 for Saline, 31.75 ± 1.49 for CNO
7F	n = 5 mice each	Paired t-test, P = 0.0651	38.47 ± 0.204 for Saline, 37.92 ± 0.216 for CNO
8B	From 3 mice, 10 sections each	N.A.	0.273 ± 0.033 for colocal / Fos, 0.178 ± 0.0167 for Colocal / mCherry
9C	n = 6 mice each	Unpaired t-test, P = 0.7842	26.58 ± 1.318 for Control, 26.13 ± 0.840 for Casp3
9D	n = 6 mice each	Unpaired t-test, P = 0.4988	888.5 ± 181.5 for Control, 730 ± 70.67 for Casp3
9E	n = 6 mice each	Unpaired t-test, P = 0.5910	37.53 ± 0.302 for Control, 37.74 ± 0.201 for Casp3
9F	n = 6 mice each	Unpaired t-test, P = 0.4422	17.69 ± 1.31 for Control, 19.66 ± 1.818 for Casp3
9G	n = 6 mice each	Unpaired t-test, P = 0.0976	22.46 ± 2.182 for Control, 33.15 ± 4.618 for Casp3
9H	n = 6 mice each	Unpaired t-test, P = 0.4187	7.50 ± 2.172 for Control, 5.667 ± 1.08 for Casp3
10E	n = 3 mice each	Fisher's exact test, P = 0.0008	N.A.

Supplementary Table 3. Detailed information of AAV construct used in this study.

Construct	Manufacture	Cat #
AAV5-hSyn-DIO-hM3D(Gq)-mCherry	Addgene	Addgene #44361
AAV1-hSyn-DIO-hM4D(Gi)-mCherry	Addgene	Addgene #44362
AAV1-hSyn-GCaMP6f-P2A-nls-dTomato	Addgene	Addgene #51085
AAV1-hSyn-FLEx-mGFP-2A-Synaptophysin-mRuby	Addgene	Addgene #71760
AAVrg/9-hSyn-Cre-WPRE-hGH	Addgene	Addgene #105553
AAV5-hSyn-DIO-mCherry	Addgene	Addgene #50459
AAV5-flex-taCasp3-TEVp	Addgene	Addgene #45580
AAV1-hSyn-hChR2(H134R)-mCherry	Addgene	Addgene #26976
AAV1/rg-CamKII-HI-GFP-Cre-WPRE-SV40	Addgene	Plasmid #105551
AAV-DJ/8-CMV-FLEX-SaCas9-U6-sgPenk-HA*<br><br>* Addgene Plasmid was packaged by institute for basic science (IBS) virus facility, Daejeon, South Korea ($10^{12.6}$ vg/mL).	Addgene / IBS*	Addgene #159916

Supplementary Table 4. Detailed information of antibody used in this study.

Antibody	Manufacture	Cat #
Rabbit anti c-Fos	AbCam	#ab190289
Rabbit anti met-enkephalin	ImmunoStar	#20065
Goat anti CGRP	AbCam	#ab36001
Donkey anti rabbit-488	Jackson ImmunoResearch	#711-545-152
Donkey anti Goat-488	Jackson ImmunoResearch	#705-545-003
Donkey anti Guinea Pig-Cy3	Jackson ImmunoResearch	#706-165-148
Donkey anti Rabbit-647	Jackson ImmunoResearch	#711-605-152
Guinea Pig anti c-Fos	SYSY	#226308
Rabbit anti HA epitope tag	Cell signaling	#3724
Rabbit anti PAX2, polyclonal	Thermofisher	#71-6000
Rabbit anti NK1R	Merck	#AB5060

Supplementary Table 5. Detailed information of FISH probe used in this study.

Probe	Manufacture	Cat #
RNAscope probe, Mm-Penk	ACD	#318761
RNAscope probe, Zsgreen-C2	ACD	#461251-C2
